# A multidimensional measure of animal ethics orientation – Developed and applied to a representative sample of the Danish public

**DOI:** 10.1371/journal.pone.0211656

**Published:** 2019-02-07

**Authors:** Thomas Bøker Lund, Sara Vincentzen Kondrup, Peter Sandøe

**Affiliations:** 1 Department of Food and Resource Economics, University of Copenhagen, Frederiksberg C, Denmark; 2 Department of Veterinary and Animal Sciences, University of Copenhagen, Frederiksberg C, Denmark; University of Portsmouth, UNITED KINGDOM

## Abstract

We present a questionnaire-based measure of four animal ethics orientations. The orientations, which were developed in light of existing empirical studies of attitudes to animal use and ethical theory, are: animal rights, anthropocentrism, lay utilitarianism, and animal protection. The two latter orientations can be viewed as variants of animal welfarism. Three studies were conducted in Denmark in order to identify the hypothesised orientations, evaluate their concurrent validity, and report their prevalence and relevance in animal-related opinion formation and behaviour. Explorative factor analysis (Study 1) and confirmative factor analysis (Study 2) successfully identified the four orientations. Study 2 revealed good measurement invariance, as there was none or very modest differential item functioning across age, gender, living area, and contrasting population segments. Evaluation of concurrent validity in Study 2 found that the orientations are associated with different kinds of behaviour and opinion when the human use of animals is involved in the hypothesised directions. In Study 3, a representative population study, the animal protection orientation proved to be most prevalent in the Danish population, and as in study 2 the four orientations were associated with different behaviours and opinions. Remarkably, the animal protection orientation does not lead to increased animal welfare-friendly meat consumption, the main reason for this being non-concern about the current welfare status of farm animals. We argue that the developed measure covers a wide range of diversity in animal ethics orientations that is likely to exist in a modern society such as Denmark and can be used in future studies to track changes in the orientations and to understand and test hypotheses about the sources and justifications of people’s animal-related opinions and behaviours.

## 1. Introduction

### 1.1. Animal ethics orientations–a framework in which animal-related opinions and behaviours can be understood

Human use of animals is becoming increasingly controversial. This can be seen in our food preferences, and specifically the consumption of meat and its alternatives, where ethically motivated vegans at one extreme avoid animal-derived food products altogether as a result of concerns about animal use [1, 2] while, at the other extreme, a significant number of people choose meat-intensive diets [3], apparently with few concerns about farm animal welfare. Between such opposing practices, consumer demand for “welfare friendly” meat has encouraged alternative animal production systems in many countries [4–6]. Of course, other areas of animal use also receive growing attention, create debate and act as spurs to action. Examples include the use of animals in scientific research and testing [7], recreational hunting [8, 9] and as companions [10]. Animal captivity in zoos [11] and the continued use of animal products such as leather and fur in clothing manufacture [12, 13] have also come under scrutiny.

Although animal use is contested in these ways, the ethical orientations that people draw on when evaluating different forms of animal use are not yet well understood. A number of “ideal”-type moral orientations have been distinguished in the literature on the ethics of animal use [14, 15]. However, with the exception of a few investigations (see Section 1.3.) research into whether such orientations play any role in the formation of opinions about, and behaviour relating to, the use of animals is largely absent. Clearly, factors other than people’s adoption of theorised ethical perspectives, which may be rather abstract and detached from everyday life, will play a part in shaping their animal-related opinions and behaviours. For example, studies have shown that strategies to avoid cognitive dissonance with respect to meat-eating are in play: the act of eating meat may reduce moral concerns for the animals eaten [16], and processes in which meat is dissociated from animals may reduce empathy towards animals and reactions of disgust at meat and meat-eating [17]. Other branches of research have correlated people’s beliefs about animal sentience with opinions about the use of animals [18]. While these factors are important, they are probably not the only determinants of animal-related opinion and behaviour, because arguably they rest upon underlying ethical beliefs. For instance, cognitive dissonance emerges only when individuals, or the cultures they participate in, recognise that their ethical assumptions about how animals should be treated have been violated.

### 1.2. Aims of this paper

The present paper aims to develop and apply a multidimensional measure of animal ethics orientations consisting of four orientations to the use of animals: “animal rights”, “anthropocentrism”, “animal protection”, and “lay utilitarianism”. The orientations were extracted from overviews of animal ethics theories in the literature [14, 15, 19] and from readings of empirical studies suggesting that some viewpoints, not precisely identified in theories of animal ethics, exist and may even be widespread in the general population. Examples of the latter are the animal protection (see Section 1.3.3.) and lay utilitarian (see Section 1.3.4.) viewpoints. In the next subsection, the four orientations are described. After this we report findings from an investigation based on data from three questionnaire studies conducted in Denmark. Our aim is to develop the multi-dimensional measure and evaluate its quality and relevance by assessing the reliability of the factorial structure, its concurrent validity, and its measurement invariance. Scores on the animal ethics orientations across socio-demographic segments in the Danish population are then reported, and we ask whether they play separate roles in the formation of animal-related opinions and behaviour in the general public in Denmark (see further detail in Section 1.4. An overview of procedures and datasets).

### 1.3. The Four ethical orientations

The four orientations offer distinctive accounts of whether, and in what way, animals matter as individual beings. They are not exhaustive, and other ethical stances capable of guiding people’s animal-related opinions and behaviour certainly exist. For instance, biospherical and environmental value orientations relate to norms about animals at the supra-individual level relating to species, populations and ecosystems [20, 21]. Eco-feminist ethics and other derivatives of postcolonial and post-humanist thinking include perspectives on race, gender, species boundaries, and transgenics, with implications for human treatment and exploitation of “non-human animals” [22, 23]. However, since these other stances do not have animal use as their primary focus, they are not considered here.

Before going into the details of the four animal ethics orientations, we review some previous studies which have also sought, on an empirical basis, to develop general measures of public attitudes to animals. These studies are general in the sense that they examine public attitudes to animals in general. Narrower studies, e.g. of the use of animals in farming [24], are not considered here. In two of these general studies [25, 26] the primary aim was to identify a single, and general, attitudinal dimension; there was no ambition to engage with different ethical orientations suggested by the theoretical literature. However, two other studies did investigate multiple domains of animal-related attitudes. Kendall and colleagues [27] identified an animal treatment scale and an animal utility scale. However, they made no attempt to distinguish between different ethical principles. Lund and colleagues [1] investigated a multidimensional animal ethics framework that distinguished between, for example, animal rights, utilitarianism, and what they called contractarianism. However, the derived sub-dimensions were correlated with each other by default, and no psychometric assessment was carried out [1].

These existing studies [1, 25–27] all employ question statements which partly, or completely, prompt respondents about specific types of animal use. In contrast, the reasoning that guided the development of items used to identify animal ethics orientations in the present study was that the questions should prompt for general values and mind-sets surrounding the four ethical lines of thought, and less so particular aspects of animal use. This meant that the resulting measures could be used to study how ethical orientations are associated with more concrete instances of behaviour and opinion relating to animal use, thereby uncovering tensions between the values people adhere to and their actual behaviour.

#### 1.3.1. Anthropocentrism

According to the *anthropocentric orientation*, human beings matter most–they are, so to speak, the centre of the moral universe. Although the orientation does not rule out that sentient non-humans have some intrinsic value and some moral standing, humans and animals are assigned different moral statuses and worth, and animals are primarily considered as means to a (human-centred) end [19]. This stance could be based on the idea that humans are intellectually superior to animals; it could also be based on the religious assumption that humans are created in God’s image; or it could be founded on the view that ethics is fundamentally a matter of self-interest, the suggestion here being that humans, unlike animals, are rational, independent individuals who are able to make agreements in their own self-interest.

#### 1.3.2. Animal rights

At the other end of the ethical spectrum the *animal rights orientation* claims that non-human sentient animals matter in the same way that humans do, and that, since humans have rights, so do sentient animals. Consequently, the animal rights orientation advocates the abolition of forms of animal use where animal interests and lives are sacrificed for the sake of other, typically human, interests [14, 15]. Animal rights proponents typically justify their position on the grounds that because animals, like humans, have certain capacities such as sentience and self-awareness, it is unacceptable to draw a moral distinction between human and non-human animals.

#### 1.3.3. Animal protection

Unlike anthropocentrism and the animal rights stance, the two remaining orientations are not established, well-described ethical theories of the sort familiar in ethical theory. Instead, they can be conceived as distinct lay views that are likely to exist among the general public. They are variations of what Garner has termed animal welfarism, or animal welfare ethics [19]. This view claims that the interests of animals matter, but unlike the animal rights orientation, animal welfarism does not question the right of humans to use animals for what are considered important human endeavours. Unlike anthropocentrism, humans have a moral obligation, as far as possible, to avoid causing suffering to animals and/or to ensure a positive quality of life for them. Essentially, a “…principle of unnecessary suffering…[is]…invoked if the level of suffering inflicted on an animal outweighs the benefits likely to be gained by humans (see [19]: 473)”.

Within this animal welfarism perspective, there is likely to be a two-fold empirical distinction in orientations, separated by the willingness to accept repugnant use of animals and severe animal suffering. This hypothesis is based on an interpretation of empirical research into lay reasoning about animal ethics (which we describe in more detail in the latter part of this section and in Section 1.3.4).

The first is what we call the *animal protection orientation*. This claims that while it is acceptable to use animals for human purposes, animals should be treated humanely and have decent lives while they are in human custody. If the welfare of the animals is threatened, actions must be taken to avoid suffering. Some form of suffering, though, may be accepted if deemed necessary. This type of ethical reasoning is arguably predominant in highly developed societies [19, 28, 29]. In theory, it underpins current legislation about the treatment of animals in modern intensive stock-farming systems in the European Union [30], even though this legislation may, in practice, allow certain treatment of animals that is difficult to defend on this stance. It has also been identified in studies of lay attitudes to the use of animals in medical research [31], the treatment of farm animals [28, 32, 33], and pest control methods that involve killing of animals [34]. The growing number of animal welfare-friendly production schemes [6] can be seen as consumer- and/or policy-driven attempts to improve the lives of farm animals within the wider assumption that animal use is not, in itself, unacceptable–the moral objections begin only when animals are not treated well, or not treated as well as they could be (cf. the principle of unnecessary suffering).

#### 1.3.4. Lay utilitarianism

The fourth orientation is what we call the *lay utilitarian orientation*. This includes a more cynical twist to the animal welfarism view. With this orientation, all forms of animal use–even those that seem morally repugnant–are in principle acceptable as long as there are corresponding human gains and these outweigh the burdens placed on the animals involved. It is even acceptable to cause animals intense pain and other forms of severe suffering just so long as the human gains are sufficiently important. This type of reasoning can be found in the formal ethical theory known as “utilitarianism” (where, unlike in the lay form of the orientation, human and animal interests are on par). In a simple form, at any rate, that theory implies that it is acceptable to use animals, or sacrifice their welfare, if the sum total of the welfare of all beings affected by the use will thereby increase [14, 15].

As suggested by Garner [19], in current practice there is an in-built assumption in this animal welfarism perspective that non-trivial human interests cancel out a utilitarian calculation whatever the cost to animals. It is therefore unlikely that, outside academia, pure and thoroughgoing utilitarianism guides lay thinking. Rather, a more intuitive version of utilitarianism is likely to exist–one in which it is taken for granted (rather than systematically assessed by weighing benefits and animal costs against each other) that humans will gain more from animal use than the animals lose, at least in particular situations or for particular purposes. Lay utilitarian reasoning has been observed, albeit indirectly, in multiple studies of animal research [35]. In this research, sub-groups of the studied populations have been shown, for example, to be ready to allow laboratory animals to endure intense suffering if the medical purpose is important enough [31]. In a similar way, studies of attitudes to pest control and wildlife management have demonstrated varying levels of acceptance of techniques that involve painful deaths [36].

### 1.4. An overview of procedures and data sets

In order to develop a multidimensional measure based on the four animal ethics orientations described in the previous paragraphs, and to provide data and analysis relevant to the aims described in Section 1.2, we conducted three studies. All procedures performed in the three studies were carried out in accordance with the ethical standards at the time of data collection. The data collection procedure for this project was furthermore approved by the Institutional Review Board at the University of Copenhagen. In the first, Study 1, we embarked on an initial examination of whether the four animal ethical orientations could be identified by examining the factorial structure that emerged from exploratory factor analysis of responses to a pool of questionnaire items given to a sample of university students.

Our aim in Study 2 was to test whether the factorial structure identified in Study 1 could be replicated in a diverse study population. We also sought to assess measurement invariance [37, 38] across socio-cultural subpopulations, and evaluate concurrent validity [39]. Measurement invariance indicates that measures of, for instance, values, attitudes, or opinions are unbiased across different cultural groups. These measures will not necessarily be invariant, because people live in culturally diverse settings and by implication are socialised differently and have separate experiences [37, 38]. Therefore, the manifest items (e.g. attitude statements) used to operationalise a measure may function differently across cultural groups: so-called differential item functioning (DIF) [38]. Items for which this is the case should be excluded to promote measurement invariance, which in turn avoids the aforementioned biased results in cross-cultural comparisons. Concurrent validity, on the other hand, is a type of criterion-related validity. It refers to whether a measure correlates with other measures (the criterion measure) as theoretically expected [39]. We found that there was not much research to draw on when we attempted to formulate expected associations between the animal ethics orientations and animal-related measures of behaviour and opinion. Nevertheless, it was possible to hypothesise a number of expected associations (these are laid out in Section 3.3).

Our goal in Study 3 was to confirm the factorial structure identified in the two previous studies in a representative sample of members of the Danish public. The prevalence of the four animal ethics orientations across age, gender, education, and living area (Danish regions) was assessed. Finally, we examined the importance of the animal ethics orientations in attitude formation and behaviour by conducting associational tests between the orientations and various types of *behaviour* that require animals to be used (e.g. visits to animal theme parks, the adoption of different types of meat-based diet, and the keeping of companion animals), a range of *opinions* (as expressed in views about two campaigns on animal welfare run in Denmark in 2017 and in the importance participants ascribed to animal welfare), and *trust* in current animal welfare legislation.

We also aimed to assess whether it is statistically justifiable to construct measures of the four latent orientations by summing the raw scores of items (ranging from 1: completely disagree to 5: completely agree) belonging to each dimension. This assessment was important, since latent variables computed on the basis of raw scores can be consistently reproduced across studies. In contrast, factor scores are always contextually bounded by the particular study sample on which the factor analysis has been conducted [40]. Scales based on sum scores facilitate cross-study comparison of, for instance, mean changes or differences in animal ethical orientations (over time and between cultures). To examine this, confirmatory factor analysis (CFA) with model restrictions corresponding to the tau-equivalent and parallel model [41] were run in Study 2 and Study 3. Non-restricted CFA, also known as the congeneric model, assesses whether each individual item belongs convincingly to one latent dimension. The tau-equivalence model assumes that items measure the latent dimension with the same degree of precision. This model can be tested by placing constraints on the factor loadings of each item. Where the tau-equivalent model holds, it is justifiable to calculate the internal consistency measure Cronbach’s alpha [41]. The parallel model assumes that all items measure the same latent scale with the same degree of precision (the equal factor loading constraint) and the same amount of error. This is tested by constraining the items to have equal error variances. If the parallel model holds, the latent scales can be calculated soundly by summing the scores of all items.

## 2. Study 1

To discover whether the four animal ethical orientations could be identified in a convenience sample of university students, we developed a pool of potential scale items (in Danish) for each of the four anticipated orientations set out in Section 1.3.

### 2.1. Sample

The questionnaire was administered at the University of Copenhagen. During two classes, on two different dates in May 2017, veterinary and food science students, respectively, were asked to fill out the questionnaire. The questionnaire was set up in an online format and students could access it through a link that was provided to them during class. To ensure anonymity, the link was the same for all students and was not in any way coupled to the students’ electronic ID at the university. There was implicit informed consent, as all students were instructed that the online data collection was completely anonymised, and that if they filled out the questionnaire they agreed that their responses would be used in subsequent data analysis and publication. This consent procedure was approved by the Institutional Review Board at the University of Copenhagen. Since nearly all university students in Denmark are at least 18 years old when they begin their studies, it was not necessary to include consent from parents/guardians. The resulting sample (N = 158) consisted of 124 (79%) veterinary and 34 (21%) food science students, including 134 (85%) women and 24 (15%) men. The average age was 24.8 (SD 3.6).

### 2.2. Materials and analysis

The first author developed an initial item pool after, which the third author then reviewed. This resulted in a revised item pool consisting of 21 statements with five response options: 1 “completely disagree”, 2 “disagree”, 3 “neither agree nor disagree”, 4 “agree”, and 5 “completely agree”. As mentioned earlier, most items prompted for general values and mind-sets connected with the human use of animals. With some of the question items–particularly those centring on the lay utilitarian orientation–we referred to a particular type of animal use, namely medical research, to ensure that respondents were cued to think about a context in which using and harming animals may be more readily justified.

In the initial phase, we conducted a principal component analysis of all 21 items in order to exclude items that did not correlate significantly with one of the four hypothesised dimensions and items that cross-loaded on several factors. Following that, 13 items remained. We evaluated these in an explorative factor analysis using MPLUS v6, using the MLR estimator, in which the standard error estimates of maximum likelihood parameters and chi^2^ test statistics are robust to non-normality. This estimator treats the questionnaire items as continuous, which is generally acceptable when there are five or more ordinal response categories [42]. We requested output with 2, 3, and 4 factors to study the improvement in fit. The factors were allowed to correlate using the default geomin (oblique) rotation, since we hypothesised that the animal protection orientation, lay utilitarian orientation, and anthropocentric orientation would be positively correlated with each other, whilst the animal rights orientation would be negatively correlated with the other three orientations. We reported chi^2^ values and the following model indices: Comparative Fit Index (CFI), Tucker-Lewis Index (TLI), and RMSEA (Root Mean Square Error of Approximation), and SRMR (Standardised Root Mean Square Residual). TLI and CFI values over .90 and RMSEA and SRMR <0.08 were considered to indicate acceptable model fit [43, 44].

### 2.3. Results

Model fit results suggest that a two-factor model provides an unsatisfactory fit to the data (CFI 0.624/TLI 0.447/RMSEA 0.202 (90%CI: 0.184–0.221)/SRMR 0.092/ Chi^2^ 395.1 (53df); p<0.001). Three-factor modelling improved model fit considerably, but in it neither CFI, TLI nor RMSEA was at an acceptable level (CFI 0.865/TLI 0.750/RMSEA 0.136 (90%CI: 0.115–0.158)/SRMR 0.059/ Chi^2^ 164.8 (42df); p<0.001). The four-factor model improved model fit further with all fit indices reaching acceptable levels (CFI .990/TLI .976/RMSEA 0.042(90%CI: 0.00–0.072)/SRMR 0.018/Chi^2^ 41.0 (32df) p>0.05). In Table 1, item statements are set out for each of the dimensions from the four-factor model along with factor loadings from the explorative factor analysis. The four animal ethics orientations (as theoretically defined in Subsections 1.3.1–1.3.4) appear to have been clearly identified in the list of items representing the factors. For instance, the items in the animal rights orientation (human use of animals “…should be prohibited by law”, “…is unacceptable because animals can feel pain, happiness, etc.”, and “…is unacceptable because animals are sentient beings”) match the moral ideas implied in this orientation. As expected, the anthropocentric, lay utilitarian, and animal protection orientation were positively correlated with one another (Avg. Pearson’s r = 0.496 (range 0.454–0.563), while the animal rights orientation was negatively correlated with them (Avg. Pearson’s r = -0.411 (range -0.343 to -0.437)).

**Table 1:** Factor structure of the multidimensional measure of animal ethics orientations—results from explorative factor analysis in Study 1 (N = 158).

	Animal rights	Anthropo-centric	Animal protection	Lay utilitarian
The use of animals by humans should be prohibited by law.	0.578			
In principle, the use of animals by humans is unacceptable because animals can feel pain, happiness, etc.	1.025			
In principle, the use of animals by humans is unacceptable because animals are sentient beings.	0.940			
We have the right to use animals because humans are intellectually superior to animals.		0.716		
Human interests are more important than those of animals.		0.921		
We must prioritize humans over animals.		0.773		
It is acceptable for humans to put animals down if it is done painlessly.			0.655	
Using animals for important human purposes (e.g. medical research) is acceptable if it is done so that the animals do not experience unnecessary stress.			0.965	
Using animals for important human purposes is acceptable if it is done so that the animals do not experience unnecessary pain.			0.945	
Using animals for important human purposes is acceptable if the animals have a decent quality of life.			0.838	
Inflicting serious pain on animals is acceptable if it is necessary in order to achieve a vital human goal–e.g. in medical research.				0.835
Inflicting considerable pain on animals is justified if the purpose is sufficiently important—e.g. medical research.				1.003
Exposing animals to stress and reducing their welfare is justified if the purpose is sufficiently important.				0.530
**Correlations between attitudinal dimensions**				
Animal rights	1.000			
Anthropocentric	-0.447	1.000		
Animal protection	-0.451	0.447	1.000	
Lay utilitarian	-0.347	0.575	0.475	1.000

Fit indices from explorative factor analysis: CFI .990/TLI .976/RMSEA 0.042/SRMR 0.018/Chi^2^ 41.0 (32df); p>0.05. Factor loadings are from geomin rotated solution. Values below 0.300 were suppressed.

### 2.4. Study 1 conclusion

Study 1 successfully identified a factorial structure corresponding to the four animal ethics orientations targeted, and all of the ethical orientations were correlated with one another in the expected directions.

## 3. Study 2

The aims were to find out whether the factorial structure identified in Study 1 could be replicated in a diverse study population, to assess measurement invariance across socio-cultural subpopulations, and to test concurrent validity. In addition to examining invariance across socio-demographic segments we examined it in two groups that are expected to be culturally and attitudinally remote: namely “meat avoiders” (i.e. vegans, vegetarians and semi-vegetarians) and people employed in the meat production sector (principally farmers, meat factory workers, butchers, and political-strategical/consultancy work in the meat sector). The recruitment of the study sample was designed so that these two groups were included along with an intermediate population of the general public in Denmark.

To examine concurrent validity, we developed a number of additional measures in the questionnaire focusing on animal-related behaviour, opinions, and dilemmas. We set up hypotheses about the particular animal ethics orientations likely to be associated with these measures. A measure of the so-called 4Ns of meat consumption (that meat is Natural, Normal, Necessary, and Nice) was reproduced from Piazza and colleagues [45], and we formulated expectations about the direction of association between endorsement of the 4Ns and the four animal ethics orientations. Similar, hypotheses were formulated about associations between the ethics orientations and the three sub-populations of the sample (meat avoiders, people employed in the meat production sector, and members of the general Danish public).

### 3.1. Sample

Respondents were recruited from a panel of Danes administered by the Danish survey company Userneeds, which hosts a panel of about 90,000 individuals living in Denmark all of whom have previously been invited in a random draw to participate in the panel. Informed consent was collected and handled by Userneeds in accordance with the rules in place at the time of data collection. The informed consent involves a two-step procedure in which individuals first agree to join the panel by actively providing their e-mail address and clicking a “Send the information” button to Userneeds. Userneeds then sends a background questionnaire to the individual. The individual only becomes a member of the panel if this questionnaire is filled out and returned. When Userneeds invited individuals to take part in this particular study, the participants also consented to this by actively clicking on a link provided by Userneeds in an email. We received the data in a completely anonymised form. This consent procedure was approved by the Institutional Review Board at the University of Copenhagen. Since the sampling scheme only invited individuals of 18 years or older, it was not necessary to include consent from parents/guardians. Respondents belonging to one of three sub-populations were invited to complete the questionnaire survey. The general public subpopulation had sampling quotas to ensure socio-demographic variation of age, gender, education and geography (i.e. Danish regions). Data was collected in July 2017. The total size of this study sample was N = 452 (Meat avoiders = 128; Employed in meat production = 104; Unspecified general population = 220). Details of the socio-demographic distribution and sub-population can be viewed in S1 Table.

### 3.2. Materials and analysis

To determine whether the factorial structure identified in Study 1 could be replicated, we conducted confirmatory factor analysis (CFA) using the MLR estimator in MPLUS, specifying which of the four factors each of the 13 items were allowed to load on to (see Table 1 for the specifications of item-to-factor belongings). The model fit evaluations used in Study 1 were again employed. To assess measurement invariance, we ran analyses of DIF against age, gender, education, region, and the three sub-populations using the hierarchical logistic regression procedure [38]. Items were flagged with DIF if the change in likelihood ratio Chi^2^ value was significant at the 0.01 level and if McKelveys and Zavoina’s pseudo-R^2^ [46] exceeded 0.035 [47]. See S1 Appendix for details. After removing items with DIF bias we re-ran CFA and evaluated the resulting four-factor model. Further, the four-factor model was run with restrictions corresponding to the tau-equivalent and parallel model [41].

In the concurrent validity test of whether the animal ethics orientations play separate roles in the formation of animal-related opinions and behaviour we ran ordinal or logit regression analyses with different opinion and stated behaviour measures (described further in section 3.3 Results) inserted as dependent variables. In all regression models the four animal ethics orientations were inserted as independent variables along with socio-demographic control variables and a variable indicating the subpopulation.

### 3.3. Results

Confirmatory factor analysis confirmed that the four-factor model provides an acceptable fit to the data (CFI .971/TLI .962/RMSEA 0.054(90%CI 0.043–0.066)/SRMR 0.039/Chi^2^ 138.1 (59df); p<0.001). There were no signs of DIF in the cases of living area, age, gender, and education (see S2–S8 Tables). However, in the three subpopulations DIF emerged for one item. This item was from the animal protection scale: “It is acceptable for humans to put animals down if it is done painlessly” operated differently among respondents from the meat production sector as compared with meat avoiders (ΔLR Chi^2^ 18.54 (2df) p<0.001/ΔR^2^ 6.2%—see S9 Table) and unspecified members of the general public (ΔLR Chi^2^10.86 (2df) p<0.01/ΔR^2^ 4.2%—see S10 Table). Therefore, the item was deleted from the scale and CFA was re-run with the remaining 12 items. In this revised modelling, the four-factor model still represented an acceptable fit to the data (CFI .973/TLI .963/ RMSEA 0.056(90%CI 0.043–0.069)/SRMR 0.036/Chi^2^ 117.0 (48df); p<0.001), and when DIF analysis for the animal protection scale was re-run in the sub-populations, no DIF was detected (see S11–S13 Tables). The four-factor tau-equivalent model (CFI .966/TLI .960/ RMSEA 0.059(90%CI 0.047–0.071)/SRMR 0.048/Chi^2^ 143.1 (56df); p<0.001) and parallel model (CFI .961/TLI .960/ RMSEA 0.059(90%CI 0.048–0.070)/SRMR 0.041/Chi^2^ 163.5 (64df); p<0.001) also provided an acceptable fit to the data (see S14 and S15 Tables for details of the factorial structure and Cronbach’s alphas). Noting that the parallel model was acceptable, we summed the raw scores from all items that belonged to the four latent dimensions and rescaled these variables to range from 0 to 100. These variables were used in the subsequent analysis of concurrent validity.

Turning to concurrent validity, we found that the four orientations are associated with different opinions and dilemmas. Further, the pattern of these associations largely accorded with our hypotheses. We expected that the animal rights orientation would be positively associated with abolitionist behaviour connected with zoos: “On principle, I do not go to zoos, because animals are kept solely for exhibition purposes”. Regression analysis confirmed this expectation (see Table 2). We expected agreeing that “It is completely fine to keep animals in zoos, as long as they thrive in captivity” to be positively associated with the animal protection orientation (this was confirmed). Agreement with this statement was also associated with the anthropocentric orientation (this was unexpected). As expected, the anthropocentric orientation was positively associated with support for a discredited and arguably humiliating use of animals: “… acceptable to use dressed, wild animals in circuses”. We expected the lay utilitarian orientation to be associated with extreme forms of trade-off between benefit and suffering, and therefore hypothesised a positive association between lay utilitarianism and agreement that “There is no reason to punish persons who have sexual intercourse with animals as long as the animal is not exposed to pain or other discomforts”. In a dilemma concerning what to do with a surplus of homeless cats we also expected that the lay utilitarian orientation would be positively associated with support for the statement “Put down the homeless cats if a new home is not quickly found, so that important resources can be used elsewhere”. Both expectations were confirmed. Additionally, the animal rights orientation was negatively associated with this response to the homeless cats dilemma (not hypothesised). Finally, we expected the lay utilitarian and the anthropocentric orientations to be associated with agreement that “There should not be limits to the use of animals in medical research”. This expectation was confirmed.

**Table 2:** Tests of association between the animal ethics dimensions and opinions about the human use of animals (N = 448).

	Animal rights	Anthropo-centric	Animal protection	Lay utili-tarian
On principle, I do not visit zoos, because animals are kept with the sole purpose of public exhibition^A^	0.024***	n.s.	n.s.	n.s.
It is completely fine to keep animals in zoos as long as they as they thrive in captivity ^A^	n.s.	0.016*	0.028***	n.s.
It is completely acceptable to show trained wild animals in circuses^A^	n.s.	0.027***	n.s.	n.s.
There is no reason to punish people who have sexual intercourse with animals as long as the animal is not exposed to pain or other discomforts ^A^	n.s.	n.s.	n.s.	0.023***
Dilemma about homeless cats: “Put down the homeless cats where a new home is not quickly found, so that important resources can be used elsewhere (reference: other responses to the dilemma”)^B^^,^^C^	-0.011*	n.s.	n.s.	0.012*
There should be no limits to the use of animals in medical research^A^	n.s.	0.022***	n.s.	0.028***

* p<0.05

** p<0.01

*** p<0.001; n.s: not significant at the 0.05 level

^A^ Results are from ordinal logit regression models (1 =“disagree; 2 =“neither agree/disagree”; 3 = agree”) where the following control variables are included: subpopulation (divided into meat avoiders, ordinary Danes, and respondents employed in the meat production sector), age, gender, education, and living area (regions of Denmark).

^B^ Introductory text to the dilemma: Many resources are used in taking care of homeless cats. It will be impossible or very difficult to find a new home for a large proportion of the cats. What do you think should be done? Other response options offered to respondents were: “I have no opinion about this”, “Let’s be more patient and only put down the cats if it is impossible to assure them proper welfare”, and “other”. They were recoded into the reference value = 0.

^C^ Results are from logit regression models Control variables were similar to the ones reported in note A.

Following Piazza and colleagues [45] we hypothesised that animal rights would be negatively associated, while the animal protection, lay utilitarian and anthropocentric orientations would be positively associated, with the 4Ns of meat consumption. These patterns were confirmed (Pearson’s r: 4N x animal rights = -0.427 (p<0.001); anthropocentric = 0.484 (p<0.001); animal protection = 0.453 (p<0.001); lay utilitarian = 0.383 (p<0.001)). We expected similar directions of association between the orientations and the sub-populations recoded according to degree of attachment to meat consumption/production (i.e. 1. meat avoiders, 2. members of the Danish public, 3. people employed in the meat production sector). This was confirmed (Spearman’s r: degree of attachment to meat consumption/production x animal rights = -0.488 (p<0.001); anthropocentric = 0.229 (p<0.001); animal protection = 0.356 (p<0.01); lay utilitarian = 0.124 (p<0.001)).

### 3.4. Study 2 conclusion

The reliability of the factorial structure identified in Study 1 was confirmed in the very diverse study sample in Study 2. Following the removal of one of the 13 items from Study 1, the remaining 12 items had no DIF issues, implying that the measure exhibits measurement invariance across socio-demographic and diverse cultural groups. The fit of the congeneric, tau-equivalent and parallel model were all satisfactory. Thus, it is justifiable to calculate the four latent variables by summing the item raw scores.

In the main, the four orientations were associated with different animal-related opinions and dilemmas as theoretically hypothesised.

## 4. Study 3

This study aimed: to confirm that the factorial structure identified in Study 1 and Study 2 could be replicated in a representative sample of Danes; to evaluate whether it is acceptable to use summated scores to construct the latent variables; to report scores on the four animal ethics orientations across socio-demographic segments in the Danish population; and to examine whether the four orientations are associated with different types of animal-related behaviour and opinion.

### 4.1. Sample

The respondents in this study were recruited from Userneeds panel of Danes used in Study 2. Informed consent was collected and handled by Userneeds in accordance with the rules in place at the time of data collection. This involved a two-step consent procedure similar to the one laid out in section 3.1. We received the data in a completely anonymised form. This consent procedure was approved by the Institutional Review Board at the University of Copenhagen. Since the sampling scheme only invited individuals of 18 years or older, it was not necessary to include consent from parents/guardians. Sampling was conducted with a view to obtaining a representative sample of Danes aged 18–75. To this end, a stratified random sample (with strata on age, region, and gender) was drawn from the panel. Data was collected in October 2018. The gross sample invited was N = 7,684 of which 1,005 completed the questionnaire, giving a 13% response rate. To assess the quality of the data, we compared the sample with census data on age, gender, education, and region (details in S16 Table). We found that the sample represented the general Danish public (aged 18–75) reasonably well on the three socio-demographic factors age, gender, and region. However, there was some misrepresentation in terms of education: the most highly educated (1½-4 years and 5 years of higher education or more) were overrepresented, while those with no education beyond compulsory school and practical education were underrepresented. To correct this misrepresentation in the subsequent analysis, we employed a weight variable in all descriptive (i.e. univariate) and bivariate analyses (weight rake factors were: education, gender, region, and age).

The data were also compared with other data sources relevant to the study’s topic (see S16 Table). We found eating behaviour (in terms of frequency of meat and vegetable/fruit intake) and political position (intended party vote at the next national parliamentary election) in the sample to be very close to the patterns observed in other surveys of the Danish population. However, the data underestimated the prevalence, in the Danish population, of annual visits to a zoo and annual visits to a circus.

### 4.2. Materials and analysis

The 12 questions that were retained from Study 2 to construct the four animal ethics orientations were presented to the respondents. We used CFA to determine whether the 12 items produced an acceptable model fit when the four factors were requested. As in Study 2, we tested the model fit of the congeneric model, the tau-equivalent model, and the parallel model [41]. We reported average scores on the four animal ethical orientations across socio-demographic variables.

To examine whether the animal ethics orientations are associated with different types of animal-related behaviours and opinions, we constructed the following measures: *Number of animal theme parks visited*, *Frequency of meat eating*, *Animal welfare-friendly meat consumption*, *Semi-vegetarianism*, *Endorsing a campaign from a Danish animal-protection NGO*, *Endorsing a campaign from the Danish farmers’ association*, *Trust in current animal husbandry legislation*, *Non-concern about animal welfare*, *cat ownership*, and *dog ownership*. Details of these measures are given in S2 Appendix. Here, it suffices to mention that the two measures concerning respondents’ endorsement of campaigns were based on actual campaigns launched in the autumn of 2017 by the largest Danish animal-protection NGO (“Dyrenes Beskyttelse”) and the Danish meat and agricultural farmers’ association (“Landbrug & Fødevarer”), respectively. The NGO campaign was critical of current pig production (see S3 Appendix). The farmers’ campaign presented a positive narrative about the welfare of Danish farm animals and invited those who were interested to visit a farm on a campaign day (see S4 Appendix).

All of these measures were employed as dependent variables in regression analyses designed to identify how the four animal ethics orientations were associated with the measures. We identified this by using a backward selection strategy in which all four orientations were initially included and then orientations were excluded one-by-one if they did not improve model fit at the 0.05 level of significance, as assessed by the likelihood ratio Chi^2^ statistic. Socio-demographic factors (gender, age, education, household type, and living area) were included as controls in all models. The particular regression model varied with the functional form of the dependent variable (linear, ordinal logistic, logistic, or poisson).

### 4.3. Results

The congeneric four-factor model provided an acceptable fit to the data (CFI .957/TLI .941/RMSEA 0.072/SRMR 0.049/ (90%CI: 0.064–0.080)/Chi^2^ 295.2 (48df); p<0.001). Model fit was reduced in the case of the tau-equivalent (CFI .938/TLI .926/RMSEA 0.080 (90%CI: 0.073–0.087)/SRMR 0.080/Chi^2^ 415.2 (56df); p<0.001) and the parallel model (CFI .921/TLI .919/RMSEA 0.084 (90%CI: 0.077–0.091)/SRMR 0.065/Chi^2^ 517.8 (64df); p<0.001). In the parallel model, the RMSEA value exceeded our pre-defined threshold (RMSEA<0.080). However, since this was a slight overrun, and all other indices were acceptable, we considered the parallel model to be overall acceptable. Therefore, the four composite scales were calculated by summating responses on all items from each dimension, and after this the dimensions were rescaled to range from 0 to 100. See S17 and S18 Tables for CFA model results.

Table 3 outlines mean scores on the four animal ethics orientations in total and across socio-demographic segments. With a mean score of 69.4 the animal protection orientation predominates in the reasoning of members of the Danish population (the other orientations run from 38.0 (lay utilitarian) to 45.8 (anthropocentric)). Women are more animal rights oriented, and less animal protection, lay utilitarian and anthropocentric oriented than men. The animal rights orientation decreases, and the animal protection orientation increases, with age, although the changes are relatively modest with negative and positive correlations around r = 0.100. The animal rights orientation decreases with higher educational levels, while scores on the anthropocentric, animal protection, and lay utilitarian orientations increase as educational level rises. The respondents’ living area (regions in Denmark) is not a distinguishing factor, although it does exhibit a weak association with the animal protection orientation.

**Table 3:** The animal ethics orientations scores in socio-demographic sectors (N = 1002–1005).

	Animal rights		Anthropocentric		Animal protection		Lay utilitarian	
	M	SD	M	SD	M	SD	M	SD
Gender								
Male	39.7	(25.5)	51.7	(23.0)	72.0	(22.5)	43.6	(25.3)
Female	48.6	(26.0)	39.7	(23.5)	66.8	(26.4)	32.2	(24.5)
*Spearmans r; p-value*	*0*.171***		*-0*.250***		*-0*.106***		*-0*.223***	
Age								
15–29 years	50.5	(26.5)	43.4	(24.7)	63.4	(27.0)	38.3	(25.8)
30–39 years	44.5	(28.7)	46.7	(26.0)	69.9	(23.1)	38.5	(28.3)
40–49 years	39.3	(25.2)	48.3	(22.9)	72.8	(20.6)	42.6	(25.6)
50–59 years	47.0	(26.5)	41.4	(25.2)	66.3	(29.3)	33.9	(25.7)
60–69 years	40.5	(23.5)	47.3	(21.1)	74.1	(21.8)	35.3	(23.4)
70 years or older	38.2	(21.8)	51.0	(21.3)	74.4	(18.9)	39.8	(20.8)
*Spearmans r; p-value*	*-0*.118***		n.s.		*0*.120***		n.s.	
Living area (regions)								
Capital region	41.7	(24.6)	46.4	(22.1)	71.3	(22.9)	37.2	(24.3)
Mid Jutland	46.5	(30.1)	47.1	(24.6)	67.9	(27.4)	39.9	(27.2)
North Jutland	46.1	(25.2)	44.5	(24.7)	66.1	(25.5)	37.4	(23.9)
Region Zealand	43.6	(25.8)	47.8	(25.4)	73.8	(21.0)	39.8	(26.7)
Southern Denmark	45.6	(26.9)	39.9	(23.4)	62.7	(28.9)	35.4	(26.7)
*p-value from F-test*	n.s.		n.s.		***		n.s.	
Education								
Cumpulsory school	50.9	(26.7)	40.4	(24.4)	56.9	(31.1)	32.5	(24.9)
High school	46.6	(26.9)	45.5	(25.4)	69.8	(23.3)	39.2	(25.4)
Vocational education	43.8	(24.8)	45.3	(23.3)	72.3	(21.0)	37.8	(23.9)
Short to medium length higher education	40.4	(25.8)	50.0	(24.9)	74.6	(19.7)	40.8	(27.6)
Long higher education	34.0	(24.8)	51.3	(23.2)	77.7	(19.5)	44.4	(26.1)
*Spearmans r; p-value*	*-0*.185***		*0*.147***		*0*.270***		*0*.130***	
Total	44.1	(26.1)	45.8	(24.0)	69.4	(24.6)	38.0	(25.5)

* p<0.05

** p<0.01

*** p<0.001

n.s: not significant at the 0.05 level

Table 4 presents results from multivariate regression analyses where all four orientations as well socio-demographic variables were inserted as explanatory variables. The orientations are associated with all of the aspects studied in connection with the Danish general public. However, their associations are different. The animal protection orientation appears to be associated primarily with mainstream animal-related behaviour such as number of animal theme parks visited and the frequency with which meat is eaten, with a stronger animal protection orientation leading to more park visits and more frequent meat eating. This is the only orientation that is associated with support for the campaign run by the Danish meat and agricultural farmers’ association (the association being positive). It is positively associated with the NGO campaign. The animal rights orientation is positively associated with semi-vegetarianism, animal welfare-friendly meat consumption, and support for the NGO campaign for improved pig welfare. Unsurprisingly, it is negatively associated with non-concern about animal welfare. The associations of the anthropocentric and lay utilitarian orientations are generally the opposite of those of the animal rights orientation. This can be seen, for example, in the case of non-concern about animal welfare. Similarly, both orientations are negatively associated with endorsement of the NGO campaign and consumption of animal welfare-friendly meat. However, the anthropocentric and lay utilitarian orientations diverge in other respects. Thus, higher levels of anthropocentrism are associated with fewer animal theme park visits, and a lower likelihood of dog ownership. Lay utilitarianism is positively associated with meat eating and negatively associated with cat ownership. Finally, it can be seen that the animal protection, anthropocentric and lay utilitarian orientations all are positively associated with trust in current animal welfare legislation applying to animal farming.

**Table 4:** Adjusted associations between animal ethics orientations and stated behaviour in areas where animals are used, having a cat or dog, and animal-related opinions and trust (N = 974–1002)–coefficients from multivariate models^A^.

	Animal rights	Anthropo-centrism	Animal protection	Lay utili-tarian
Number of animal theme parks visited^B^	n.s.	-0.06**	0.009***	n.s.
Frequency of meat eating^C^	n.s.	n.s.	0.006***	0.004**
Animal welfare-friendly meat consumption^D^	0.016***	-0.11**	n.s.	-0.11***
Semi-vegetarianism^E^	0.015**	n.s.	n.s.	n.s.
Cat ownership^E^	n.s.	n.s.	-0.09*	-0.09*
Dog ownership^E^	n.s.	-0.14***	n.s.	n.s.
Trust in current animal welfare legislation^D^	n.s.	0.031***	0.010***	0.014***
Endorsing an NGO animal welfare campaign^C^	0.005***	-0.005**	0.004*	-0.08***
Endorsing a campaign from the Danish meat and agricultural farmers’ association^C^	n.s.	n.s.	0.08***	n.s.
Non-concern about animal welfare^C^	-0.003**	0.016***	n.s.	0.010***

* p<0.05

** p<0.01

*** p<0.001; n.s: not significant at the 0.05 level

^A^ Control variables in all analyses were: gender, age, household type (single adult, two adults (no children), and household with children), educational level, and living area.

^B^ Reported coefficients are from a Poisson regression (suitable when the dependent variable is a count variable).

^C^ Reported coefficients are from ols regression.

^D^ Reported coefficients are from an ordinal logit regression.

E Reported coefficients are from a logit regression.

The beta coefficients from multivariate analyses in Table 4 do not specify the strength of the associations between the orientations and different behaviours, opinions, and levels of trust. Therefore, we present unadjusted correlation coefficients in Table 5. It can be seen that the orientations are quite strongly associated with non-concern about animal welfare and trust in current legislation (with correlation coefficients in the medium-to-large effect size interval, i.e. r>0.300 [48]) and somewhat less strongly with animal welfare-friendly meat consumption and endorsing an NGO animal welfare campaign (correlation coefficients in the 0.200–0.300 interval). Noticeably weaker correlation coefficients characterise frequency of meat eating, cat/dog ownership, semi-vegetarianism, and number of theme parks visited.

**Table 5:** Unadjusted correlation coefficients between animal ethics orientations and stated behaviour in areas where animals are used, having a cat or dog, and animal-related opinions and trust (N = 974–1002).

	Animal rights	Anthropo-centrism	Animal protection	Lay utili-tarian
Number of animal theme parks visited^A^	-0.0321^n.s.^	-0.0354^n.s.^	0.0791**	-0.0237^n.s.^
Frequency of meat eating^B^	-0.0819^n.s.^	0.1986***	0.1752***	0.2083***
Animal welfare-friendly meat consumption^C^	0.2706***	-0.265***	-0.2216***	-0.2815***
Semi-vegetarianism^C^	0.1547***	-0.0991***	-0.1468***	-0.0842***
Cat ownership^C^	0.0918**	-0.1082***	-0.1214***	-0.1101***
Dog ownership^C^	0.1046***	-0.1195***	-0.0611^n.s.^	-0.1056***
Trust in current animal welfare legislation^C^	-0.2435***	0.4707***	0.3009***	0.4064***
Endorsing an NGO animal welfare campaign^B^	0.2388***	-0.2795***	-0.1632***	-0.2934***
Endorsing a campaign from the Danish meat and agricultural farmers’ association^B^	-0.0147 ^n.s.^	0.0222 ^n.s.^	0.1235***	0.0286 ^n.s.^
Non-concern about animal welfare^B^	-0.3122***	0.6013***	0.3367***	0.5625***

* p<0.05

** p<0.01

*** p<0.001; n.s: not significant at the 0.05 level

^A^ Pearson’s r coefficients. P-values are from a Poisson regression.

^B^ Pearson’s r coefficients and p-values.

^C^ Spearman’s rho coefficient and p-values.

Table 5 also shows that all orientations are associated with the studied behaviours and opinions before adjusting for the other orientations. As such, the orientations can be seen as interrelated perspectives conveying adjacent principles of evaluation. Particular orientations are more powerful in specific opinion formation processes and behaviours, as shown by the adjusted results in Table 4.

#### 4.3.1 Animal protection orientation and animal welfare-friendly meat consumption–a follow-up examination

Remarkably, Table 5 also shows that the animal protection orientation, the most predominant orientation among members of the Danish public, is negatively associated with animal welfare-friendly meat consumption (spearman’s r = -0.2216) before adjustment. Intuitively, the opposite would have been expected. Furthermore, as argued in the Introduction (section 1.3.3), the ideology behind current legislation regarding the treatment of animals in modern intensive stock-farming systems (for instance in Europe [30]) is largely inspired by an animal welfare ethics view that animals should not experience unnecessary suffering [19], i.e. an animal protection orientation. So this unexpected finding requires further explanation.

One possibility is that people with an animal protection orientation believe that current animal welfare legislation is adequate. This makes animal welfare-friendly meat consumption superfluous. Another possibility is that the animal protection-orientated are simply not particularly concerned about animal welfare, at least when it comes to putting their money where their mouth is. To examine these competing explanations we performed mediation analysis. This kind of analysis disentangles how much of the effect from an explanatory variable (x) on a dependent variable (y) is confounded by a number of intervening variables (z). Since we had an ordered outcome variable we used the method proposed by Breen and colleagues [49], an approach which circumvents the problem that logit coefficients from nested models are not directly comparable [50]. We inserted animal welfare-friendly meat consumption as outcome (y), and animal protection orientation as key variable (x). We inserted the following five mediator variables (z), trust in current animal welfare legislation, non-concern about animal welfare, and the three remaining animal ethics orientations, i.e. animal rights, anthropocentrism, and lay utilitarianism.

From Table 6, it can be seen that these five variables mediate a very large proportion (83.5%) of the association between the animal protection orientation and animal welfare-friendly meat consumption. Non-concern about animal welfare is the mediator variable accounting for the largest proportion of the association (41.3%). Trust in current legislation mediates a relatively small part of the association (13.5%). Among the ethics orientations, the animal rights orientation is the only one to mediate the association to a relatively large extent, accounting for 27.9%.

**Table 6:** Decomposition of the extent to which five variables (Animal rights, Anthropocentrism, Lay utilitarian, Non-concern, and Trust in current animal welfare legislation) mediate the effect of the Animal protection orientation on Animal welfare-friendly meat consumption (N = 976)^A^^,^^B^.

		Confounding (%)
Animal protection (variable of interest that is decomposed)		
Reduced (unadjusted model)	-0.0264***	
Full model (adjusted model)	-0.004^n.s.^	
Difference (summary of confounding)	-0.022***	83.5
Components of difference		
Animal rights	-0.0073	27.92
Anthropocentrism	0.0016	-5.98
Lay utilitarian	-0.0018	6.73
Non-concern about animal welfare	-0.0109	41.32
Trust in current animal welfare legislation	-0.0035	13.55

* p<0.05

** p<0.01

*** p<0.001; n.s: not significant at the 0.05 level

^A^ Decomposition based on the khb method outlined by Breen, Karlson, & Holm, A. (2013) and implemented in Stata’s khb package.

^B^ Control variables included (using the concomitant command in the khb package) were: gender, age, household type (single adult, two adults (no children), and household with children), educational level, and living area.

In sum, then, the mediation analysis suggests that non-concern about animal welfare is the main reason the animal protection orientation is negatively associated with animal welfare-friendly meat consumption.

### 4.4. Study 3 conclusion

The factorial structure identified in Study 1 and Study 2 was confirmed in this representative sample of Danish citizens. Further, the fit of the tau-equivalence and parallel model turned out to be acceptable. The results suggest that a number of socio-demographic differences (e.g. gender, age, and education) affect the distribution of the four animal ethics orientations in Denmark. The orientations are also associated with different types of animal-related behaviour and opinion in the Danish population. The strength of these associations varies, with opinion measures (such as non-concern and trust in legislation) being most strongly associated with the orientations.

## 5. Discussion

We set out to develop a measure of animal ethics orientations based on the hypothesised existence of four orientations. The orientations were identified and confirmed in three studies where explorative and confirmatory factor analysis was undertaken. We found that the orientations can be identified using 12 questionnaire items. At most, these items show very modest signs of DIF bias across a range of socio-demographic factors, implying measurement invariance. Further, we found that the four orientations can reasonably be constructed into variables by summing the raw scores of the factor-specific items. This makes the four orientations easy to reproduce and facilitates cross-study comparison of mean scores [40].

One item with DIF was identified in Study 2, namely “It is acceptable for humans to put animals down if it is done painlessly”. Although item bias was modest, this finding suggests that attitudes to the putting down of animals constitute a separate dimension within the wider animal welfarism framework laid out by Garner [19]. This possibility could be usefully examined in future studies.

The assessments of concurrent validity (Study 2) were in general satisfactory, as the expected associations between animal ethics orientations and opinions, behaviours, and trust were largely confirmed. It should be noted that no prior studies could be utilised as a gold standard for this assessment. Still, in Study 2 and Study 3 it was found that the four animal ethics orientations are associated with distinctively different groups of animal-related opinion and behaviour. This supports our initial assumption that it is important to differentiate the ethical domains.

Study 3, which featured a representative sample of members of the Danish public, suggested that the animal ethics orientations are at least partly determined by the same socio-structural factors as those observed in a separate representative study [27], conducted in the US, where latent scales with somewhat overlapping meaning were employed. The US study, which surveyed a representative population sample (households in Ohio), found patterns in gender and education similar to those we observed: women and those with fewer years of education were more likely to attach importance to the proper treatment of animals and less likely to exhibit utilitarian attitudes. Kendall and colleagues refer to this pattern as an “underdog” mechanism, meaning by this that population segments which historically, or currently, have fewer resources and lower status tend to sympathise more with animals as a result of their powerless position in society [27]. Since we found similar patterns–with women/lower educated having higher animal rights scores, while men/higher educated had higher animal protection, anthropocentric and lay utilitarian scores–this phenomenon is probably cross-cultural, as also hypothesised by Kendall and colleagues [27].

The perspective added by the multidimensional measure employed here can be seen in its ability to detect the type of ethical orientation that is influential in various types of behaviour requiring animals to be used, and in opinions. From previous research, it was known that animal rights are extremely important for vegetarians and vegans [e.g. 1, 2]. In line with this, we found animal rights to be associated with semi-vegetarianism and the consumption of animal welfare-friendly meat. We have added to the existing body of knowledge by showing that, animal rights, in contrast, has little bearing on general frequency of meat eating or whether animal parks are visited. More mainstream behaviours of this kind are instead positively associated with our newly developed sub-scale: the animal protection orientation.

Non-concern about animal welfare was found to be much higher among highly anthropocentric and lay utilitarian individuals, who were also more willing to accept relatively controversial use of animals, including the clothing of animals in circuses, and widely condemned activities such as human-animal sexual intercourse. Interestingly, the ownership of a dog, on the one hand, and the ownership of a cat, on the other, seem to be associated with different animal ethics orientations, as the likelihood of having a dog decreases with stronger anthropocentric views, while the likelihood of having a cat decreased with higher lay utilitarian and animal protection scores. This is an unexpected finding which deserves further investigation.

In the case of dog ownership, we speculate that there may be two possible explanations for the negative correlation with anthropocentric views: anthropocentric people, who are probably relatively uninterested in engaging with animals (recall the negative association here with animal park visits), may not be motivated to embark on the resource-heavy and time-consuming responsibilities dog ownership involves. Conversely, over time people who do own a dog may develop less anthropocentric attitudes as a consequence of their experiences with, and attachment to, the dog.

We have not been able to come up with a convincing explanation as to why cat ownership is associated with higher levels of lay utilitarian and animal protection orientations. This is something that deserves further study.

In line with the interpretation of the animal protection orientation as a mainstream sentiment, we found that it was the only orientation to be (positively) associated with a positive attitude to the campaign run by the Danish meat and agricultural farmers’ association. As shown above, the animal protection orientation was not associated with animal welfare-friendly meat consumption in the adjusted analysis (Table 4) and was negatively correlated with animal welfare-friendly meat consumption in unadjusted analysis (Table 5). This is peculiar, since the orientation stresses that the welfare of animals is important in its own right, and that animals must be treated humanely and without unnecessary suffering. The central question is: If the animal protection orientation does not operate as a “light” version of, or mainstream correction to, the more radical animal rights orientation, at least in Denmark, then what drives this orientation, and the negative association with animal welfare-friendly meat consumption?

We found that the primary explanation of the negative association was a lack of concern about animal welfare in contemporary society. It appears, then, that people who are strongly animal-protection oriented can distance themselves from the animal welfare consequences of meat eating [32, 51] by allowing them to argue there are more important issues on the political agenda than animal welfare. This finding supports observations that even though the animal welfarism surrounding modern societies assigns some moral worth to animals, it nevertheless facilitates the continued use of animals, including use involving suffering, stress or similar [14, 19, 29]. It will be important in future to research the pattern of association between the animal protection orientation and animal welfare-friendly meat consumption over time and in other countries–not least because there is an ongoing controversy among scholars as to whether an animal rights or a more pragmatic animal protection-orientated position should be promoted in the public sphere in an effort to improve the lives of animals [52].

The measure was developed in Denmark, and as a result it has to be asked whether it would be equally sound in other national or regional settings. The factorial structure should be re-examined in studies featuring other populations. However, since the orientations in this study were developed on the basis of ethical theories and empirical studies from multiple countries, we believe it is likely that the orientations will be found in other countries as well.

The main empirical contribution of the measure lies in its capacity to deliver richer and more nuanced empirical studies of the attitudinal sources and justifications of different forms of animal use. Furthermore, to our knowledge, this is the first study to attempt to empirically assess the forms that the animal welfarism framework takes in real life [19], and to separate it from classical animal ethics viewpoints (i.e. anthropocentrism and animal rights).We also believe it can be used to unveil tensions between people’s values and what they actually do. It is a remarkable and ironic finding, for example, that a stronger animal protection orientation does not make people more likely to consume animal welfare-friendly meat. As we have noted, there was even a negative correlation here in the unadjusted analysis. Future studies could test hypotheses about this, drawing, for instance, on cognitive dissonance theory [16] and the effects of categorizing animals as food [53], as other studies of meat consumption have done. It is also important to understand whether this pattern is culturally specific to Denmark, as a country with a long history of intensive animal farming. Cross-country historical institutional differences may very well affect the process of public attitude formation on this point.

In general, we believe that the developed measure covers a wide range of diversity of animal ethics orientations in a modern society like Denmark. It can be used in future studies to make cross-cultural comparisons and track changes in the orientations and understand and test hypotheses about the sources and justifications of people’s animal-related opinions and behaviours.

## Supporting information

S1 Table Subpopulation and socio-demographic characteristics of Study 2 (N = 452). (DOCX)

S2 Table Test of Differential Item Functioning between Regions in Denmark—East (capital city of Copenhagen and Zealand and Southern Islands) and West Denmark (Fünen and Jutland and Islands) compared (N = 452). (DOCX)

S3 Table Test of Differential Item Functioning between Capitol Region in Denmark and remaining parts of Denmark (N = 452). (DOCX)

S4 Table Test of Differential Item Functioning between age groups–youngest (18–34 years) and oldest (66 years or more) compared (N = 225). (DOCX)

S5 Table Test of Differential Item Functioning between age groups–middle aged (35–65 years) and oldest (66 years or more) compared (N = 321). (DOCX)

S6 Table Test of Differential Item Functioning between age groups–middle aged (35–65 years) and youngest (18–35 years) compared (N = 358). (DOCX)

S7 Table Test of Differential Item Functioning between educational groups–low (compulsory, high school, short tertiary education) and high (medium length or long tertiary education) compared (N = 452). (DOCX)

S8 Table Test of Differential Item Functioning between sub-populations–meat avoiders (i.e. semi-vegetarian, vegetarian, vegan) and unspecified members of the general Danes population (i.e. non vegetarian and not employed in meat sector related occupations) compared (N = 348). (DOCX)

S9 Table Test of Differential Item Functioning between sub-populations–meat avoiders (i.e. semi-vegetarian, vegetarian, vegan) and people employed in the meat production sector (farmers/farm workers, consultants/strategic work, butchers, slaughter-related work) compared (N = 232). (DOCX)

S10 Table Test of Differential Item Functioning between sub-populations–unspecified members of the general Danes population and people employed in meat production sector compared (N = 324). (DOCX)

S11 Table Test of Differential Item Functioning between sub-populations after modification of the Animal Protection scale–meat avoiders and people employed in meat production sector compared (N = 232). (DOCX)

S12 Table Test of Differential Item Functioning between sub-populations after modification of the Animal Protection scale–unspecified members of the general Danes population and people employed in meat production sector compared (N = 324). (DOCX)

S13 Table Test of Differential Item Functioning between sub-populations after modification of the Animal Protection scale–unspecified members of the general Danes population and meat avoiders compared (N = 348). (DOCX)

S14 Table CFA model results after removal of the item “It is acceptable for humans to put animals down if it is done painlessly” from the animal protection orientation (N = 452). (DOCX)

S15 Table Factor structure of the multidimensional measure of animal ethics orientations—results from final parallel model (with 12 items) in Study 2 (N = 452). (DOCX)

S16 Table Study 3 sample descriptive characteristics and comparison with population census (Danes aged 18–75 years) and other data sources on eating behavior and political party inclination. (DOCX)

S17 Table CFA model results from Study 3 (N = 1005). (DOCX)

S18 Table Factor structure of the multidimensional measure of animal ethics orientations—results from final parallel model Study 3 (N = 1005). (DOCX)

S1 Appendix Analysis of differential item functioning (an overview). (DOCX)

S2 Appendix Measures used in Study 3 (N = 1005). (DOCX)

S3 Appendix Campaign from Danish animal-protection NGO (“Dyrenes Beskyttelse”). (DOCX)

S4 Appendix Campaign from Danish meat and agricultural farmers’ association (“Landbrug & Fødevarer”). (DOCX)

S5 Appendix Questionnaire used in Study 1. (DOCX)

S6 Appendix Questionnaire used in Study 2. (DOCX)

S7 Appendix Questionnaire used in Study 3. (DOCX)

S1 Data Codebook Study 1. (CSV)

S2 Data Data from Study 1. (CSV)

S3 Data Codebook Study 2. (CSV)

S4 Data Data from Study 2. (CSV)

S5 Data Codebook Study 3. (CSV)

S6 Data Data from Study 3. (CSV)
