# Supplementary material for: A multidimensional measure of animal ethics orientation – Developed and applied to a representative sample of the Danish public
Source: PLoS One. 2019 Feb 7;14(2):e0211656. doi: 10.1371/journal.pone.0211656 (PMC6366885; doi:10.1371/journal.pone.0211656)
Supplement: S1 Table — (DOCX) [file pone.0211656.s001.docx]

|  | |
| --- | --- |
|  | N |
| **Subpopulation details^a^** |  |
| Meat avoiders | 128 |
| Semi-vegetarian | (47) |
| Vegetarian | (51) |
| Vegan | (30) |
| Employed in meat production^b^ | 104 |
| Farmer/farm worker | (66) |
| Consultant/adviser in animal husbandry, slaughter houses and meat processing | (22) |
| Strategic, economic, political work on animal husbandry | (7) |
| Butcher, slaughter worker, animal slaughter transport, meat processing company | (13) |
| The general public | 220 |
| Socio-demographic factors |  |
| **Gender** |  |
| Male | 210 |
| Female | 242 |
| **Age (years)** |  |
|  | 131 |
|  | 100 |
| 50-65 | 127 |
| 66-80 | 94 |
| **Education^c^** |  |
| Compulsory school | 55 |
| High school | 47 |
| Vocational education | 118 |
| Short tertiary education (1-2 years) | 31 |
| Medium length tertiary education (>2-4 years) | 112 |
| Long tertiary education (>=5 years) | 85 |
| **Geographical area (Region)^c,d^** |  |
| Capital region | 365 |
| Mid Jutland region | 83 |
| North Jutland region | 37 |
| Region Zealand | 61 |
| Region of Southern Denmark | 106 |
| Notes: ^a^ Respondents that both were meat avoiders and workers in meat production were classified as meat avoider; ^b^ The total sums to more than 100, as this was a multiple response question | |
