# Supplementary material for: A multidimensional measure of animal ethics orientation – Developed and applied to a representative sample of the Danish public
Source: PLoS One. 2019 Feb 7;14(2):e0211656. doi: 10.1371/journal.pone.0211656 (PMC6366885; doi:10.1371/journal.pone.0211656)
Supplement: S7 Table — (DOCX) [file pone.0211656.s007.docx]

|  | | | | | | | | | | | | |
| --- | --- | --- | --- | --- | --- | --- | --- | --- | --- | --- | --- | --- |
| *Animal Rights Items* | | | | | | | | | | | | |
|  | The use of animals by humans should be prohibited by law | | | In principle, the use of animals by humans is unacceptable because animals can feel pain, happiness, etc. | | | In principle, the use of animals by humans is unacceptable because animals are sentient beings. | | |  | | |
| Test statistics | Uniform DIF | Total DIF | Non-uniform DIF | Uniform DIF | Total DIF | Non-uniform DIF | Uniform DIF | Total DIF | Non-uniform DIF |  |  |  |
| ∆ Chi^2^ | 0,69 | 0,71 | 0,02 | 0,13 | 0,18 | 0,05 | 0,01 | 2,13 | 2,12 |  |  |  |
| p-value | n.s. | n.s. | n.s. | n.s. | n.s. | n.s. | n.s. | n.s. | n.s. |  |  |  |
| ∆ R^2^ | 0,001 | 0,001 | 0,000 | 0,000 | 0,000 | 0,000 | 0,000 | 0,002 | 0,002 |  |  |  |
| *Animal Protection Items* | | | | | | | | | | | | |
|  | It is acceptable for humans to put animals down if it is done painlessly. | | | Using animals for important human purposes (e.g. medical research) is acceptable if it is done so that the animals do not experience unnecessary stress. | | | Using animals for important human purposes is acceptable if it is done so that the animals do not experience unnecessary pain. | | | Using animals for important human purposes is acceptable if the animals have a decent quality of life. | | |
| Test statistics | Uniform DIF | Total DIF | Non-uniform DIF | Uniform DIF | Total DIF | Non-uniform DIF | Uniform DIF | Total DIF | Non-uniform DIF | Uniform DIF | Total DIF | Non-uniform DIF |
| ∆ Chi^2^ | 0,30 | 1,89 | 1,59 | 0,50 | 2,66 | 2,16 | 0,47 | 2,23 | 1,76 | 0,05 | 1,15 | 1,11 |
| p-value | n.s. | n.s. | n.s. | n.s. | n.s. | n.s. | n.s. | n.s. | n.s. | n.s. | n.s. | n.s. |
| ∆ R^2^ | 0,001 | 0,002 | 0,001 | 0,001 | 0,002 | 0,002 | 0,000 | 0,002 | 0,002 | 0,000 | 0,002 | 0,002 |
| *Lay Utilitarian Items* | | | | | | | | | | | | |
|  | Inflicting serious pain on animals is acceptable if it is necessary in order to achieve a vital human goal – e.g. in medical research. | | | Inflicting considerable pain on animals is justified if the purpose is sufficiently important - e.g. medical research. | | | Exposing animals to stress and reducing their welfare is justified if the purpose is sufficiently important. | | |  | | |
| Test statistics | Uniform DIF | Total DIF | Non-uniform DIF | Uniform DIF | Total DIF | Non-uniform DIF | Uniform DIF | Total DIF | Non-uniform DIF |  |  |  |
| ∆ Chi^2^ | 2,84 | 3,80 | 0,96 | 0,18 | 0,29 | 0,10 | 0,30 | 0,38 | 0,08 |  |  |  |
| p-value | n.s. | n.s. | n.s. | n.s. | n.s. | n.s. | n.s. | n.s. | n.s. |  |  |  |
| ∆ R^2^ | 0,002 | 0,003 | 0,001 | 0,000 | 0,000 | 0,000 | 0,000 | 0,000 | 0,000 |  |  |  |
| *Anthropocentric Items* | | | | | | | | | | | | |
|  | We have the right to use animals because humans are intellectually superior to animals. | | | Human interests are more important than those of animals. | | | We must prioritize humans over animals. | | |  | | |
| Test statistics | Uniform DIF | Total DIF | Non-uniform DIF | Uniform DIF | Total DIF | Non-uniform DIF | Uniform DIF | Total DIF | Non-uniform DIF |  |  |  |
| ∆ Chi^2^ | 17,69 | 17,70 | 0,01 | 13,65 | 19,03 | 5,38 | 2,05 | 2,62 | 0,57 |  |  |  |
| p-value | ** | ** | n.s. | ** | ** | * | n.s. | n.s. | n.s. |  |  |  |
| ∆ R^2^ | 0,017 | 0,017 | 0,000 | 0,008 | 0,002 | 0,002 | 0,002 | 0,002 | 0,000 |  |  |  |
| ** p<0.01; * p<0.05; n.s. not significant at the 0,05 level | | | | | | | | | | | | |
