# Supplementary material for: A multidimensional measure of animal ethics orientation – Developed and applied to a representative sample of the Danish public
Source: PLoS One. 2019 Feb 7;14(2):e0211656. doi: 10.1371/journal.pone.0211656 (PMC6366885; doi:10.1371/journal.pone.0211656)
Supplement: S9 Table — (DOCX) [file pone.0211656.s009.docx]

|  | | | | | | | | | | | | |
| --- | --- | --- | --- | --- | --- | --- | --- | --- | --- | --- | --- | --- |
| *Animal Rights Items* | | | | | | | | | | | | |
|  | The use of animals by humans should be prohibited by law | | | In principle, the use of animals by humans is unacceptable because animals can feel pain, happiness, etc. | | | In principle, the use of animals by humans is unacceptable because animals are sentient beings. | | |  | | |
| Test statistics | Uniform DIF | Total DIF | Non-uniform DIF | Uniform DIF | Total DIF | Non-uniform DIF | Uniform DIF | Total DIF | Non-uniform DIF |  |  |  |
| ∆ Chi^2^ | 5,06 | 5,78 | 0,72 | 0,88 | 3,36 | 2,48 | 2,57 | 15,23 | 12,67 |  |  |  |
| p-value | * | n.s. | n.s. | n.s. | n.s. | n.s. | n.s. | ** | ** |  |  |  |
| ∆ R^2^ | 0,008 | 0,013 | 0,005 | 0,001 | 0,002 | 0,002 | 0,002 | 0,015 | 0,013 |  |  |  |
| *Animal Protection Items* | | | | | | | | | | | | |
|  | It is acceptable for humans to put animals down if it is done painlessly. | | | Using animals for important human purposes (e.g. medical research) is acceptable if it is done so that the animals do not experience unnecessary stress. | | | Using animals for important human purposes is acceptable if it is done so that the animals do not experience unnecessary pain. | | | Using animals for important human purposes is acceptable if the animals have a decent quality of life. | | |
| Test statistics | Uniform DIF | Total DIF | Non-uniform DIF | Uniform DIF | Total DIF | Non-uniform DIF | Uniform DIF | Total DIF | Non-uniform DIF | Uniform DIF | Total DIF | Non-uniform DIF |
| ∆ Chi^2^ | 13,98 | 18,54 | 4,56 | 2,09 | 4,89 | 2,80 | 0,90 | 9,32 | 8,42 | 0,01 | 2,27 | 2,26 |
| p-value | ** | ** | ***** | n.s. | n.s. | n.s. | n.s. | ** | ** | n.s. | n.s. | n.s. |
| ∆ R^2^ | **0,031** | **0,062** | **0,031** | 0,006 | 0,004 | 0,004 | 0,003 | 0,011 | 0,008 | -0,001 | 0,001 | 0,002 |
| *Lay Utilitarian Items* | | | | | | | | | | | | |
|  | Inflicting serious pain on animals is acceptable if it is necessary in order to achieve a vital human goal – e.g. in medical research. | | | Inflicting considerable pain on animals is justified if the purpose is sufficiently important - e.g. medical research. | | | Exposing animals to stress and reducing their welfare is justified if the purpose is sufficiently important. | | |  | | |
| Test statistics | Uniform DIF | Total DIF | Non-uniform DIF | Uniform DIF | Total DIF | Non-uniform DIF | Uniform DIF | Total DIF | Non-uniform DIF |  |  |  |
| ∆ Chi^2^ | 3,93 | 4,01 | 0,08 | 1,77 | 2,40 | 0,63 | 0,82 | 1,62 | 0,80 |  |  |  |
| p-value | * | n.s. | n.s. | n.s. | n.s. | n.s. | n.s. | n.s. | n.s. |  |  |  |
| ∆ R^2^ | 0,005 | 0,005 | 0,000 | 0,002 | 0,001 | 0,001 | 0,001 | 0,002 | 0,001 |  |  |  |
| *Anthropocentric Items* | | | | | | | | | | | | |
|  | We have the right to use animals because humans are intellectually superior to animals. | | | Human interests are more important than those of animals. | | | We must prioritize humans over animals. | | |  | | |
| Test statistics | Uniform DIF | Total DIF | Non-uniform DIF | Uniform DIF | Total DIF | Non-uniform DIF | Uniform DIF | Total DIF | Non-uniform DIF |  |  |  |
| ∆ Chi^2^ | 0,28 | 1,08 | 0,80 | 0,43 | 0,44 | 0,01 | 0,97 | 1,27 | 0,30 |  |  |  |
| p-value | n.s. | n.s. | n.s. | n.s. | n.s. | n.s. | n.s. | n.s. | n.s. |  |  |  |
| ∆ R^2^ | 0,001 | 0,007 | 0,006 | 0,001 | 0,000 | 0,000 | 0,001 | 0,001 | 0,000 |  |  |  |
| ** p<0.01; * p<0.05; n.s. not significant at the 0,05 level | | | | | | | | | | | | |
