# Supplementary material for: A multidimensional measure of animal ethics orientation – Developed and applied to a representative sample of the Danish public
Source: PLoS One. 2019 Feb 7;14(2):e0211656. doi: 10.1371/journal.pone.0211656 (PMC6366885; doi:10.1371/journal.pone.0211656)
Supplement: S13 Table — (DOCX) [file pone.0211656.s013.docx]

|  | | | | | | | | | | | | |
| --- | --- | --- | --- | --- | --- | --- | --- | --- | --- | --- | --- | --- |
| *Animal Protection Items* | | | | | | | | | | | | |
|  | ITEM REMOVED | | | Using animals for important human purposes (e.g. medical research) is acceptable if it is done so that the animals do not experience unnecessary stress. | | | Using animals for important human purposes is acceptable if it is done so that the animals do not experience unnecessary pain. | | | Using animals for important human purposes is acceptable if the animals have a decent quality of life. | | |
| Test statistics |  |  |  | Uniform DIF | Total DIF | Non-uniform DIF | Uniform DIF | Total DIF | Non-uniform DIF | Uniform DIF | Total DIF | Non-uniform DIF |
| ∆ Chi^2^ |  |  |  | 2,26 | 10,62 | 8,36 | 0,02 | 3,24 | 3,22 | 0,70 | 0,75 | 0,05 |
| p-value |  |  |  | n.s. | ** | ** | n.s. | n.s. | n.s. | n.s. | n.s. | n.s. |
| ∆ R^2^ |  |  |  | 0,002 | 0,008 | 0,006 | 0,000 | 0,002 | 0,002 | 0,001 | 0,001 | 0,000 |
