# Supplementary material for: A multidimensional measure of animal ethics orientation – Developed and applied to a representative sample of the Danish public
Source: PLoS One. 2019 Feb 7;14(2):e0211656. doi: 10.1371/journal.pone.0211656 (PMC6366885; doi:10.1371/journal.pone.0211656)
Supplement: S14 Table — (DOCX) [file pone.0211656.s014.docx]

|  | | | | | | | | |
| --- | --- | --- | --- | --- | --- | --- | --- | --- |
|  | Chi2 | df | p-value | CFI | TLI | RMSEA | (90% CI) | SRMR |
| Congeneric model | 117.0 | 48 | <0.000 | 0.973 | 0.963 | 0.056 | (0.043-0.069) | 0.036 |
| Tau-equivalent model | 143.1 | 56 | <0.000 | 0.966 | 0.960 | 0.059 | (0.047-0.071) | 0.046 |
| Parallel model | 163.5 | 64 | <0.000 | 0.961 | 0.960 | 0.059 | (0.048-0.070) | 0.048 |
