# Supplementary material for: A multidimensional measure of animal ethics orientation – Developed and applied to a representative sample of the Danish public
Source: PLoS One. 2019 Feb 7;14(2):e0211656. doi: 10.1371/journal.pone.0211656 (PMC6366885; doi:10.1371/journal.pone.0211656)
Supplement: S15 Table — (DOCX) [file pone.0211656.s015.docx]

|  | | | | |
| --- | --- | --- | --- | --- |
|  | Animal Rights | Anthropocentric | Animal  Protection | Lay Utilit-arian |
| The use of animals by humans should be prohibited by law | 0.854 |  |  |  |
| In principle, the use of animals by humans is unacceptable because animals can feel pain, happiness, etc. | 0.854 |  |  |  |
| In principle, the use of animals by humans is unacceptable because animals are sentient beings. | 0.854 |  |  |  |
| We have the right to use animals because humans are intellectually superior to animals. |  | 0.858 |  |  |
| Human interests are more important than those of animals. |  | 0.858 |  |  |
| We must prioritize humans over animals. |  | 0.858 |  |  |
| Using animals for important human purposes (e.g. medical research) is acceptable if it is done so that the animals do not experience unnecessary stress. |  |  | 0.859 |  |
| Using animals for important human purposes is acceptable if it is done so that the animals do not experience unnecessary pain. |  |  | 0.859 |  |
| Using animals for important human purposes is acceptable if the animals have a decent quality of life. |  |  | 0.859 |  |
| Inflicting serious pain on animals is acceptable if it is necessary in order to achieve a vital human goal – e.g. in medical research. |  |  |  | 0.783 |
| Inflicting considerable pain on animals is justified if the purpose is sufficiently important - e.g. medical research. |  |  |  | 0.783 |
| Exposing animals to stress and reducing their welfare is justified if the purpose is sufficiently important. |  |  |  | 0.783 |
| *Cronbach’s alpha* | *0.890* | *0.893* | *0.894* | *0.826* |
| **Correlations between attitudinal dimensions** |  |  |  |  |
| Animal Rights | 1.000 |  |  |  |
| Anthropocentric | -0.507 | 1.000 |  |  |
| Animal Protection | -0.706 | 0.563 | 1.000 |  |
| Lay Utilitarian | -0.415 | 0.855 | 0.495 | 1.000 |
