# Supplementary material for: A multidimensional measure of animal ethics orientation – Developed and applied to a representative sample of the Danish public
Source: PLoS One. 2019 Feb 7;14(2):e0211656. doi: 10.1371/journal.pone.0211656 (PMC6366885; doi:10.1371/journal.pone.0211656)
Supplement: S16 Table — (DOCX) [file pone.0211656.s016.docx]

|  | | |
| --- | --- | --- |
|  | Population census | Public sample |
| **Gender** |  |  |
| Man | 50.2% | 50% |
| Woman | 49.8% | 50% |
| **Age** |  |  |
| 15-29 years | 21.8% | 18.4% |
| 30-39 years | 16.1% | 14.8% |
| 40-49 years | 18.5% | 21.5% |
| 50-59 years | 18.8% | 12.7% |
| 60-69 years | 15.9% | 21.1% |
| >69 years | 8.8% | 11.4% |
| **Education** |  |  |
| No education | 23.1% | 7.2% |
| High school | 10.8% | 13.7% |
| Practical education | 35.1% | 23.5% |
| 1-4½ years higher education | 22.0% | 37.8% |
| 5 years higher education or more | 8.8% | 17.8% |
| **Geographical area (Region)** |  |  |
| Capital Region | 31.9% | 31.8% |
| Region Zealand | 14.4% | 13.4% |
| Region of Southern Denmark | 21.0% | 22.2% |
| Mid Jutland region | 22.5% | 23.8% |
| North Jutland region | 10.2% | 8.8% |
| **Eating behavior^A^** | Other data source | Study 3 sample^B^ |
| Eats meat 1-2 times per day or more | 35.0% | 28.0% |
| Eats fish 1-2 times per day or more | 11.7% | 10.9% |
| Eats warm dish with meat (beef, pork, veal) 5-7 days per week or more | 22.6% | 18.9% |
| Eats warm dish with poultry 5-7 days per week or more | 7.1% | 7.4% |
| Eats warm dish with fish 5-7 days per week or more | 3.4% | 3.8% |
| Eats vegetable or vegetarian dish 5-7 days per week or more | 16.0% | 15.1% |
| **Going to theme parks (last year)^C^** |  |  |
| Zoo or other animal theme park | 42.0% | 30.8% |
| Circus | 17.7% | 4.1% |
| Aquariums | 17.6% | 13.8% |
| **Intended political party vote^D^** |  |  |
| Socialdemokratiet | 26.4% | 23.8% |
| Radikale Venstre | 5.8% | 5.4% |
| Det Konservative Folkeparti | 4.2% | 5.2% |
| Nye Borgerlige | 1.5% | 1.5% |
| Socialistisk Folkeparti | 4.8% | 8.2% |
| Danske Folkeparti | 18.2% | 17.7% |
| Alternativet | 5.2% | 5.3% |
| Venstre | 18.7% | 15.8% |
| Liberal Alliance | 6.8% | 5.7% |
| Enhedslisten | 8.6% | 9.3% |
| Other | 1% | 0.5% |
| ^A^ Data comparison source is “National Health Interview Survey”. Data was collected in 2013 (see SUSY database at <http://www.si-folkesundhed.dk/Forskning/Befolkningens%20sundhedstilstand/Sundhed%20og%20sygelighed%20SUSY/SUSY-databaser.aspx>). The comparison data was provided to us from Ola Ekholm, chief statistician in the Danish National Health Interview Survey  ^B^ Weighted percentages  ^C^ Data comparison source is “The Danes' Participation in Cultural and Leisure Activities 2012, adults” (Bak et al, 2012). | | |
| ^D^ Data comparison source is the weighted average of multiple political party polls as calculated on 7^th^ October 2017 by Berlingske Barometer (http://www.politiko.dk/barometeret). Response to the question “Which party would you vote for, if there were a governmental election [Folketingsvalg] tomorrow?” | | |
