# Supplementary material for: A multidimensional measure of animal ethics orientation – Developed and applied to a representative sample of the Danish public
Source: PLoS One. 2019 Feb 7;14(2):e0211656. doi: 10.1371/journal.pone.0211656 (PMC6366885; doi:10.1371/journal.pone.0211656)
Supplement: S17 Table — (DOCX) [file pone.0211656.s017.docx]

|  | | | | | | | | |
| --- | --- | --- | --- | --- | --- | --- | --- | --- |
|  | Chi2 | df | p-value | CFI | TLI | RMSEA | (90% CI) | SRMR |
| Congeneric model | 295.2 | 48 | <0.000 | 0.957 | 0.941 | 0.072 | (0.064-0.080) | 0.049 |
| Tau-equivalent model | 415.2 | 56 | <0.000 | 0.938 | 0.926 | 0.080 | (0.073-0.087) | 0.080 |
| Parallel model | 517.8 | 64 | <0.000 | 0.921 | 0.919 | 0.084 | (0.077-0.091) | 0.065 |
