# Supplementary material for: A multidimensional measure of animal ethics orientation – Developed and applied to a representative sample of the Danish public
Source: PLoS One. 2019 Feb 7;14(2):e0211656. doi: 10.1371/journal.pone.0211656 (PMC6366885; doi:10.1371/journal.pone.0211656)
Supplement: S1 Appendix — (DOCX) [file pone.0211656.s019.docx]

Tests of differential functioning was conducted using the ordinal logistic regression DIF method described in detail in Zumbo [1]. This involves a hierarchical regression procedure where each of the manifest (ordinal) item (denoted as *i* below) belonging to a latent variable is inserted as dependent variable in four nested ordinal regression models. Using the notation from Choi, Gibbons and Crane [2], *u_i_* (= 0, 1,…,m_1_ - 1) denotes the response to item *i* with *m_i_* ordered response categories. Using the parallel regression assumption a set of regression coefficients with varying intercepts (*a_k_*) is estimated for all cumulative logits.

Model 0*:* Logit *P(u_i_ ≥ k) = a_k_*

Model 1*:* Logit *P(u_i_ ≥ k) = a_k_ + β_1_*latent_variable*

Model 2*:* Logit *P(u_i_ ≥ k) = a_k_ + β_1_*latent_variable + β_2_*group*

Model 3*:* Logit *P(u_i_ ≥ k) = a_k_ + β_1_*latent_variable + β_2_*group + β_3_*latent_variable*group*

The *group* variable denotes the background factor that is being assessed for measurement invariance (in this analysis: geographical location, gender, age, education, and sub-population). The *latent_variable* denotes the latent dimension the item is postulated to belong to. In this analysis there are four latent variables, namely Animal Rights, Animal Welfare, Lay Utilitarian, and Anthropocentric. For each dimension there are three (and in the case of Animal welfare, four) items that are inserted as candidate items in the hierarchical regression procedure.

Items exhibit DIF if the model fit of the data improves when the group variable is inserted into the equation as assessed by changes in likelihood ratio chi^2^ values and pseudo-R^2^. An overall evaluation of “total DIF-effect” can be done by comparing Model 1 and Model 3 (with 2 degrees of freedom in the likelihood ratio test), uniform DIF can be evaluated by comparing Model 1 and Model 2 (1 degree of freedom), while non-uniform DIF can be evaluated by comparing Model 2 and Model 3 (1 degree of freedom).

Following the logic in Zumbo [1], an item was flagged for DIF if the total DIF-effect (referred to as “Total DIF” in S2 – S13 Tables) showed that: the likelihood ratio test is significant at the 0.01 level and that the magnitude of the differential item functioning is high. There have been different suggestions as to what constitutes a substantial magnitude. We follow the relatively conservative suggestion by Jodoin and Gierl [3] and set the threshold if the change in R^2^ is >=0.035 (using McKelveys and Zavoina’s pseudo-R^2^) [4]. “Uniform” and “Non-uniform DIF” were also reported in S2 – S13 Tables to assess the particular form of item bias. However, we primarily conclude on basis of the “Total DIF” evaluation.

**References**

1. Zumbo BD. A handbook on the theory and methods of differential item functioning (DIF): Logistic regression modeling as a unitary framework for binary and Likert-type (ordinal) item scores. Ottawa, Canada: Directorate of Human Resources Research and Evaluation, Department of National Defense; 1999.

2. Choi SW, Gibbons LE, Crane PK. lordif: An R Package for Detecting Differential Item Functioning Using Iterative Hybrid Ordinal Logistic Regression/Item Response Theory and Monte Carlo Simulations. J Stat Softw. 2011; 39: 1-30.

3. Jodoin MG, Gierl MJ. Evaluating Type I Error and Power Rates Using an Effect Size Measure with the Logistic Regression Procedure for DIF Detection. Appl Meas Educ. 2010; 14: 329-349.

4. McKelvey RD, Zavoina W. A statistical model for the analysis of ordinal level dependent variables. J Math Sociol. 1975; 4: 103-112.
