# Supplementary material for: A multidimensional measure of animal ethics orientation – Developed and applied to a representative sample of the Danish public
Source: PLoS One. 2019 Feb 7;14(2):e0211656. doi: 10.1371/journal.pone.0211656 (PMC6366885; doi:10.1371/journal.pone.0211656)
Supplement: S2 Appendix — (DOCX) [file pone.0211656.s020.docx]

*Number of animal theme parks visited:* this is a count variable (range 0-3) summarising how many types of animal theme park (options were: zoos, circuses, aquariums) were visited the last year.

*Frequency of meat eating:* this is a continuous variable (M=0, SD=1) based on factor scores calculated from a principal component analysis of responses to three food frequency questions focusing on the intake of meat: meat fillings (response options 1: “never/seldom eat this” to 5: “twice a day”), warm dishes with meat (beef, pork, veal), and warm dishes with poultry (response options in the latter two items: 1: “never/seldom eat this” to 5: “once a day”). In combination, the three questions comprise an acceptable underlying measure of meat intake frequency (avg. Pearson’s r=0.352; Cronbach’s alpha=0.61).

*Animal welfare-friendly meat consumption:* this is based on the statement “I attempt to eat meat solely from farm production schemes where there is extra emphasis on animal welfare” (response options: 1 “Completely disagree” to 5. “Completely agree”).

*Semi-vegetarianism:* this is a binary variable (0=not a semi-vegetarian (reference value); 1=semi-vegetarian) based on whether the respondent characterises him or herself as “A person who generally eats vegetarian foods, but sometimes, i.e. 1-2 times a week or on special occasions, eats meat”

*Endorsing an NGO animal welfare campaign:* This is a continuous variable (M=0, SD=1) based on factor scores calculated from a principal component analysis of responses to three questions related to the following campaign: In the early autumn of 2017 the largest Danish animal protection NGO (Dyrenes Beskyttelse) launched a PR campaign using advertising signboards and national newspapers which focused on improvement of the welfare of Danish production pigs. We presented respondents with a poster from this campaign (see S3 Appendix for details) and showed respondents three statements: “I have great sympathy for the campaign”, “The message in the campaign is misleading (reverse coded)”, and “The campaign is good because it says something central about the way pigs are treated in Danish farms”. The response options were: 1 “Completely disagree” to 5. “Completely agree”. In combination, the three questions comprise a very acceptable underlying measure of campaign endorsement (avg. Pearson’s r=0.570; Cronbach’s alpha=0.80).

*Endorsing a campaign from the Danish meat and agricultural farmers’ association:* This is a continuous variable (M=0, SD=1) based on factor scores calculated from a principal component analysis of responses to three questions related to the following campaign: In the early autumn of 2017 the Danish farmers association (Landbrug & Fødevarer) also launched a PR campaign using advertising signboards and national newspapers (see Supplementary material G for details). This campaign focused on and highlighted the relatively high welfare of Danish farm animals, compared with other European countries. It also encouraged readers of the campaign to visit a Danish farm on a designated campaign date. We presented respondents with a poster from this campaign (see S4 Appendix for details) and showed respondents three statements: “I have great sympathy for the campaign”, “The message in the campaign is misleading (reverse coded)”, and “The campaign is good because it says something central about the way pigs are treated in Danish farms”. The response options were: 1 “Completely disagree” to 5. “Completely agree”. In combination, the three questions comprise a very acceptable underlying measure of campaign endorsement (avg. Pearson’s r=0.502; Cronbach’s alpha=0.75).

*Trust in current animal welfare legislation:* this consists of one statement: “The existing legislation is sufficient to ensure that animals used to produce meat and dairy have a decent life” (response options: 1 “Completely disagree” to 5. “Completely agree”).

*Non-concern about animal welfare:* this is a continuous variable (M=0, SD=1) based on factor scores calculated from a principal component analysis of responses to three statements focusing on the opinion that animal welfare receives too much attention and that other things are of greater concern: “In my view, all this talk about animal welfare is exaggerated”, “The focus on animal welfare is too much of a fashion fad”, and “Society has more important things to think about than the welfare of animals” (response options: 1 “Completely disagree” to 5. “Completely agree”). In combination, the three questions comprise an acceptable underlying measure of non-concern (avg. Pearson’s r=0.706; Cronbach’s alpha=0.88).

*Cat ownership:* respondents were asked whether there are cats in their households (0=no cat (reference value); 1=cat(s)).

*Dog ownership:* respondents were asked whether there are dogs in their households (0=no dog(reference value); 1=dog(s)).
