# Supplementary material for: A multidimensional measure of animal ethics orientation – Developed and applied to a representative sample of the Danish public
Source: PLoS One. 2019 Feb 7;14(2):e0211656. doi: 10.1371/journal.pone.0211656 (PMC6366885; doi:10.1371/journal.pone.0211656)
Supplement: S3 Appendix — (DOCX) [file pone.0211656.s021.docx]

In the online questionnaire, the respondents in study 3 were introduced to a poster from the Danish NGO *Dyrenes Beskyttelse*. In the table below, the original Danish poster is shown in the left hand column, while an English translation is provided in the right hand column.

| **ORIGINAL POSTER (presented to respondents)** | **ENGLISH TRANSLATION** |
| --- | --- |
|  | **Name of the NGO**  **IF ONLY PIGS WERE HENS**  You have said no to battery hens – now the pig needs your help  BATTERY **NO**  PIGS **THANKS** |
