# Supplementary material for: A multidimensional measure of animal ethics orientation – Developed and applied to a representative sample of the Danish public
Source: PLoS One. 2019 Feb 7;14(2):e0211656. doi: 10.1371/journal.pone.0211656 (PMC6366885; doi:10.1371/journal.pone.0211656)
Supplement: S4 Appendix — (DOCX) [file pone.0211656.s022.docx]

In the online questionnaire, the respondents in study 3 were introduced to a poster from the Danish farmers’ association (Landbrug & Fødevarer). In the table below, the original Danish poster is shown in the left hand column, while an English translation is provided in the right hand column.

| **ORIGINAL POSTER (presented to respondents)** | **ENGLISH TRANSLATION** |
| --- | --- |
| 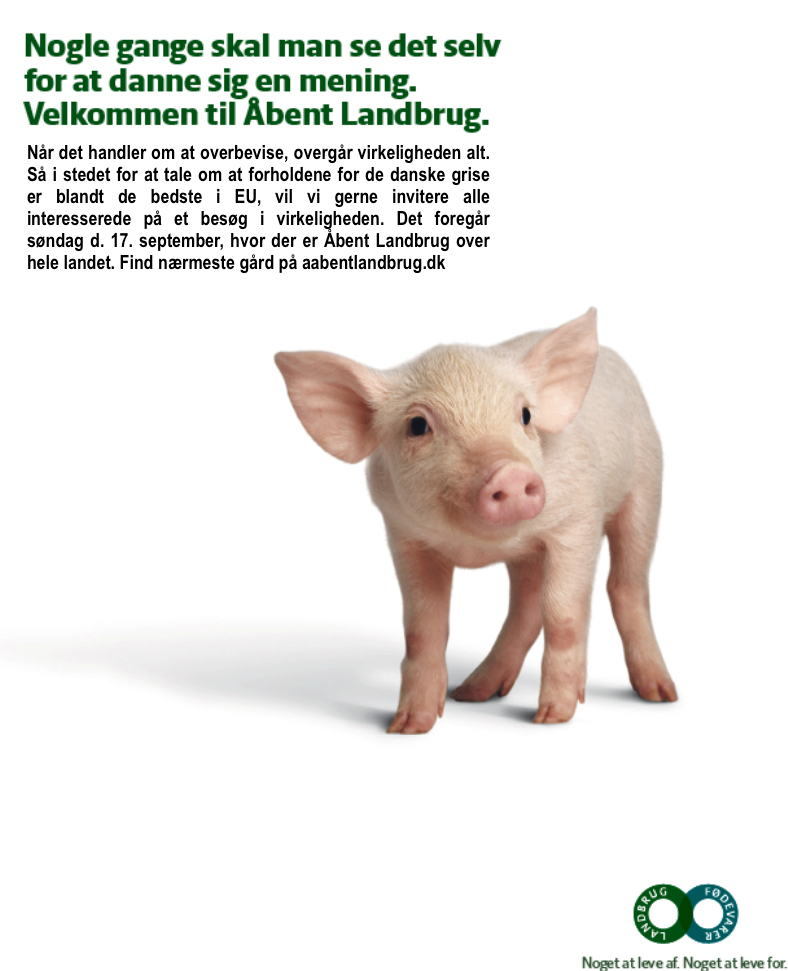 | **Sometimes you have to see it for yourself to form an attitude. Welcome to Open Farm.**  When it comes to persuading, reality trumps everything else. So instead of just telling you that the condition of pigs in Denmark is among the best in the EU, we would like to invite you to a visit to reality. Throughout the country, we have Open Farm on Sunday 17^th^ September. Find your nearest farm on aabenlandbrug.dk.  **Farm and Food** |
