# Supplementary material for: A multidimensional measure of animal ethics orientation – Developed and applied to a representative sample of the Danish public
Source: PLoS One. 2019 Feb 7;14(2):e0211656. doi: 10.1371/journal.pone.0211656 (PMC6366885; doi:10.1371/journal.pone.0211656)
Supplement: S5 Appendix — (DOCX) [file pone.0211656.s023.docx]

| Question / spørgsmål | Response category / svarmulighed | Filter / filter |
| --- | --- | --- |
| 1. Are you a student? | Single Response  1. Yes  2. No | Everyone |
| 1. Er du studerende? | Enkelt svarmulighed  1. Ja  2. Nej | Alle |
| 2. If you are a student, what are you studying? | Open-ended response | Everyone |
| 2. Hvis Ja, hvad studerer du? | Åben svarmulighed | Alle |
| 3. What is your age? | Open-ended response | Everyone |
| 3. Hvad er din alder? | Åben svarmulighed | Alle |
| 4. What is your gender? | Single Response  1. Female  2. Male | Everyone |
| 4. Hvad er din køn? | Enkelt svarmulighed  1. Ja  2. Nej | Alle |
| 5. Animals should have the same rights as humans. | Single Response  1. Completely disagree  2. Disagree  3. Neither agree nor disagree  4. Agree  5. Completely agree | Everyone |
| 5. Dyr bør have de samme rettigheder som mennesker. | Enkelt svarmulighed  1. Helt uenig  2. Uenig  3. Hverken enig eller uenig  4. Enig  5. Helt enig | Alle |
| 6. There is broad support for the idea of equal human rights – now it is time to introduce animal rights. | Single Response  1. Completely disagree  2. Disagree  3. Neither agree nor disagree  4. Agree  5. Completely agree | Everyone |
| 6. Der er bred tilslutning til ideen om lige menneskerettigheder – det er nu på tide at introducere dyrerettigheder. | Enkelt svarmulighed  1. Helt uenig  2. Uenig  3. Hverken enig eller uenig  4. Enig  5. Helt enig | Alle |
| 7. There are no good arguments in support of the human use of animals. | Single Response  1. Completely disagree  2. Disagree  3. Neither agree nor disagree  4. Agree  5. Completely agree | Everyone |
| 7. Det er ikke nogen gode argumenter for, at vi mennesker bruger dyr. | Enkelt svarmulighed  1. Helt uenig  2. Uenig  3. Hverken enig eller uenig  4. Enig  5. Helt enig | Alle |
| 8. The use of animals by humans should be prohibited by law. | Single Response  1. Completely disagree  2. Disagree  3. Neither agree nor disagree  4. Agree  5. Completely agree | Everyone |
| 8. Det burde forbydes ved lov, at mennesker bruger dyr. | Enkelt svarmulighed  1. Helt uenig  2. Uenig  3. Hverken enig eller uenig  4. Enig  5. Helt enig | Alle |
| 9. Euthanasia of animals is only acceptable when they are severely ill or injured. | Single Response  1. Completely disagree  2. Disagree  3. Neither agree nor disagree  4. Agree  5. Completely agree | Everyone |
| 9. Det er kun acceptabelt at aflive dyr, når de er alvorligt syge eller tilskadekomne. | Enkelt svarmulighed  1. Helt uenig  2. Uenig  3. Hverken enig eller uenig  4. Enig  5. Helt enig | Alle |
| 10. In principle, the use of animals by humans is unacceptable because animals can feel pain, happiness, etc. | Single Response  1. Completely disagree  2. Disagree  3. Neither agree nor disagree  4. Agree  5. Completely agree | Everyone |
| 10. Det er som udgangspunkt uacceptabelt at mennesker bruger dyr, fordi dyr kan føle smerte, glæde og lignende. | Enkelt svarmulighed  1. Helt uenig  2. Uenig  3. Hverken enig eller uenig  4. Enig  5. Helt enig | Alle |
| 11. In principle, the use of animals by humans is unacceptable because animals are sentient beings. | Single Response  1. Completely disagree  2. Disagree  3. Neither agree nor disagree  4. Agree  5. Completely agree | Everyone |
| 11. Det er som udgangspunkt uacceptabelt at mennesker bruger dyr, fordi dyr er væsner, som sanser og tænker. | Enkelt svarmulighed  1. Helt uenig  2. Uenig  3. Hverken enig eller uenig  4. Enig  5. Helt enig | Alle |
| 12. We have the right to use animals but when we do so, they should not experience severe pain. | Single Response  1. Completely disagree  2. Disagree  3. Neither agree nor disagree  4. Agree  5. Completely agree | Everyone |
| 12. Vi mennesker har ret til at bruge dyr, men når vi gør det, må de ikke opleve væsentlig smerte. | Enkelt svarmulighed  1. Helt uenig  2. Uenig  3. Hverken enig eller uenig  4. Enig  5. Helt enig | Alle |
| 13. It is okay to keep animals in captivity as long as they are treated properly. | Single Response  1. Completely disagree  2. Disagree  3. Neither agree nor disagree  4. Agree  5. Completely agree | Everyone |
| 13. Det er i orden at holde dyr i fangenskab, hvis blot de behandles ordentligt. | Enkelt svarmulighed  1. Helt uenig  2. Uenig  3. Hverken enig eller uenig  4. Enig  5. Helt enig | Alle |
| 14. We have the right to keep animals in captivity if we make sure that their needs are met (expressed by, for example, the 5 Freedoms: freedom from hunger and thirst; discomfort; pain, injury, or disease; fear and distress; freedom to express (most) normal behaviour. | Single Response  1. Completely disagree  2. Disagree  3. Neither agree nor disagree  4. Agree  5. Completely agree | Everyone |
| 14. Vi mennesker har ret til at holde dyr i fangenskab, hvis vi sikrer os, at deres behov bliver opfyldt (fx udtrykt ved de 5 friheder: frihed fra: sult/ tørst, ubehag, smerte/skade/sygdom, angst/frygt, frihed til at udtrykke normal adfærd). | Enkelt svarmulighed  1. Helt uenig  2. Uenig  3. Hverken enig eller uenig  4. Enig  5. Helt enig | Alle |
| 15. It is acceptable to kill animals if it serves a decent purpose. | Single Response  1. Completely disagree  2. Disagree  3. Neither agree nor disagree  4. Agree  5. Completely agree | Everyone |
| 15. Det er acceptabelt at slå dyr ihjel, hvis det tjener et ordentligt formål. | Enkelt svarmulighed  1. Helt uenig  2. Uenig  3. Hverken enig eller uenig  4. Enig  5. Helt enig | Alle |
| 16. It is acceptable for humans to put animals down if it is done painlessly. | Single Response  1. Completely disagree  2. Disagree  3. Neither agree nor disagree  4. Agree  5. Completely agree | Everyone |
| 16. Det er acceptabelt for mennesker at aflive dyr, hvis det foregår på en måde, så de ikke oplever smerte. | Enkelt svarmulighed  1. Helt uenig  2. Uenig  3. Hverken enig eller uenig  4. Enig  5. Helt enig | Alle |
| 17. Using animals is acceptable as long as we make sure that they experience good welfare. | Single Response  1. Completely disagree  2. Disagree  3. Neither agree nor disagree  4. Agree  5. Completely agree | Everyone |
| 17. Det er acceptabelt, at vi bruger dyr, så længe vi sørger for, at dyrenes oplever god velfærd. | Enkelt svarmulighed  1. Helt uenig  2. Uenig  3. Hverken enig eller uenig  4. Enig  5. Helt enig | Alle |
| 18. Using animals for important human purposes (e.g. medical research) is acceptable if it is done so that the animals do not experience unnecessary stress. | Single Response  1. Completely disagree  2. Disagree  3. Neither agree nor disagree  4. Agree  5. Completely agree | Everyone |
| 18. Det er acceptabelt at bruge dyr til vigtige menneskelige formål (f.eks. medicinsk forskning), hvis det sikres at dyrene ikke oplever unødig stress. | Enkelt svarmulighed  1. Helt uenig  2. Uenig  3. Hverken enig eller uenig  4. Enig  5. Helt enig | Alle |
| 19. Using animals for important human purposes is acceptable if it is done so that the animals do not experience unnecessary pain. | Single Response  1. Completely disagree  2. Disagree  3. Neither agree nor disagree  4. Agree  5. Completely agree | Everyone |
| 19. Det er acceptabelt at bruge dyr til vigtige menneskelige formål, hvis det sikres at dyrene ikke oplever unødig smerte. | Enkelt svarmulighed  1. Helt uenig  2. Uenig  3. Hverken enig eller uenig  4. Enig  5. Helt enig | Alle |
| 20. Using animals for important human purposes is acceptable if the animals have a decent quality of life. | Single Response  1. Completely disagree  2. Disagree  3. Neither agree nor disagree  4. Agree  5. Completely agree | Everyone |
| 20. Det er acceptabelt at bruge dyr til vigtige menneskelige formål, hvis dyrene har et ordentligt liv. | Enkelt svarmulighed  1. Helt uenig  2. Uenig  3. Hverken enig eller uenig  4. Enig  5. Helt enig | Alle |
| 21. Animal suffering and joy are equal to human suffering and joy. | Single Response  1. Completely disagree  2. Disagree  3. Neither agree nor disagree  4. Agree  5. Completely agree | Everyone |
| 21. Dyrs lidelse og glæde tæller lige så meget som menneskers lidelse og glæde. | Enkelt svarmulighed  1. Helt uenig  2. Uenig  3. Hverken enig eller uenig  4. Enig  5. Helt enig | Alle |
| 22. It is important to focus on the overall animal welfare at group or herd level. | Single Response  1. Completely disagree  2. Disagree  3. Neither agree nor disagree  4. Agree  5. Completely agree | Everyone |
| 22. Det er vigtigt at fokusere på den samlede velfærd i gruppen eller flokken af dyr. | Enkelt svarmulighed  1. Helt uenig  2. Uenig  3. Hverken enig eller uenig  4. Enig  5. Helt enig | Alle |
| 23. Inflicting serious pain on animals is acceptable if it is necessary in order to achieve a vital human goal – e.g. in medical research. | Single Response  1. Completely disagree  2. Disagree  3. Neither agree nor disagree  4. Agree  5. Completely agree | Everyone |
| 23. Det kan være acceptabelt at påføre dyr alvorlig smerte, hvis det er nødvendigt for at opnå et vitalt menneskeligt formål – fx i medicinsk forskning. | Enkelt svarmulighed  1. Helt uenig  2. Uenig  3. Hverken enig eller uenig  4. Enig  5. Helt enig | Alle |
| 24. Using animals is only acceptable if it benefits people more than it does damage to the animals used. | Single Response  1. Completely disagree  2. Disagree  3. Neither agree nor disagree  4. Agree  5. Completely agree | Everyone |
| 24. Det er kun acceptabelt at bruge dyr, hvis det gavner mennesker mere, end det gør skade på de dyr, der bruges. | Enkelt svarmulighed  1. Helt uenig  2. Uenig  3. Hverken enig eller uenig  4. Enig  5. Helt enig | Alle |
| 25. Inflicting considerable pain on animals is justified if the purpose is sufficiently important - e.g. medical research. | Single Response  1. Completely disagree  2. Disagree  3. Neither agree nor disagree  4. Agree  5. Completely agree | Everyone |
| 25. Det kan godt forsvares at påføre dyr væsentlig smerte, hvis formålet er vigtigt nok (f.eks. medicinsk forskning). | Enkelt svarmulighed  1. Helt uenig  2. Uenig  3. Hverken enig eller uenig  4. Enig  5. Helt enig | Alle |
| 26. Exposing animals to stress and reducing their welfare is justified if the purpose is sufficiently important. | Single Response  1. Completely disagree  2. Disagree  3. Neither agree nor disagree  4. Agree  5. Completely agree | Everyone |
| 26. Det kan godt forsvares at dyr oplever stress og velfærdsforringelser, hvis formålet er vigtigt nok. | Enkelt svarmulighed  1. Helt uenig  2. Uenig  3. Hverken enig eller uenig  4. Enig  5. Helt enig | Alle |
| 27. There is no reason to punish persons who have sexual intercourse with animals as long as the animal is not exposed to pain or other discomforts. | Single Response  1. Completely disagree  2. Disagree  3. Neither agree nor disagree  4. Agree  5. Completely agree | Everyone |
| 27. Der er ingen grund til at straffe seksuel omgang med dyr, hvis dyret ikke bliver udsat for smerte eller andet ubehag. | Enkelt svarmulighed  1. Helt uenig  2. Uenig  3. Hverken enig eller uenig  4. Enig  5. Helt enig | Alle |
| 28. Euthanasia of surplus animals in zoos is acceptable if it serves to maintain the welfare of the remaining animals. | Single Response  1. Completely disagree  2. Disagree  3. Neither agree nor disagree  4. Agree  5. Completely agree | Everyone |
| 28. Det er acceptabelt at aflive overskudsdyr i zoologiske haver, hvis det tjener til at sikre velfærden af de resterende dyr. | Enkelt svarmulighed  1. Helt uenig  2. Uenig  3. Hverken enig eller uenig  4. Enig  5. Helt enig | Alle |
| 29. We have the right to use animals. | Single Response  1. Completely disagree  2. Disagree  3. Neither agree nor disagree  4. Agree  5. Completely agree | Everyone |
| 29. Vi mennesker har ret til at bruge dyr. | Enkelt svarmulighed  1. Helt uenig  2. Uenig  3. Hverken enig eller uenig  4. Enig  5. Helt enig | Alle |
| 30. Using animals is part of the order of nature. | Single Response  1. Completely disagree  2. Disagree  3. Neither agree nor disagree  4. Agree  5. Completely agree | Everyone |
| 30. Det er en del af naturens orden, at mennesker bruger dyr. | Enkelt svarmulighed  1. Helt uenig  2. Uenig  3. Hverken enig eller uenig  4. Enig  5. Helt enig | Alle |
| 31. Human interests are more important than those of animals. | Single Response  1. Completely disagree  2. Disagree  3. Neither agree nor disagree  4. Agree  5. Completely agree | Everyone |
| 31. Menneskehedens interesser er vigtigere end dyrs interesser. | Enkelt svarmulighed  1. Helt uenig  2. Uenig  3. Hverken enig eller uenig  4. Enig  5. Helt enig | Alle |
| 32. We have the right to use animals because humans are intellectually superior to animals. | Single Response  1. Completely disagree  2. Disagree  3. Neither agree nor disagree  4. Agree  5. Completely agree | Everyone |
| 32. Vi har ret til at bruge dyr, fordi mennesker er intellektuelt overlegne i forhold til dyrene. | Enkelt svarmulighed  1. Helt uenig  2. Uenig  3. Hverken enig eller uenig  4. Enig  5. Helt enig | Alle |
| 33. We have the right to use animals regardless of the consequences for the animals. | Single Response  1. Completely disagree  2. Disagree  3. Neither agree nor disagree  4. Agree  5. Completely agree | Everyone |
| 33. Vi har ret til at bruge dyr, uanset konsekvenserne for dyrene. | Enkelt svarmulighed  1. Helt uenig  2. Uenig  3. Hverken enig eller uenig  4. Enig  5. Helt enig | Alle |
| 34. We must prioritize humans over animals. | Single Response  1. Completely disagree  2. Disagree  3. Neither agree nor disagree  4. Agree  5. Completely agree | Everyone |
| 34. Vi skal tage hensyn til mennesker frem for dyr. | Enkelt svarmulighed  1. Helt uenig  2. Uenig  3. Hverken enig eller uenig  4. Enig  5. Helt enig | Alle |
| 35. I believe that cheaper food is more important than animal welfare. | Single Response  1. Completely disagree  2. Disagree  3. Neither agree nor disagree  4. Agree  5. Completely agree | Everyone |
| 35. Jeg mener, at billigere fødevarer er vigtigere end dyrevelfærd. | Enkelt svarmulighed  1. Helt uenig  2. Uenig  3. Hverken enig eller uenig  4. Enig  5. Helt enig | Alle |
| 36. There should be no restrictions on the use of animals for medical research. | Single Response  1. Completely disagree  2. Disagree  3. Neither agree nor disagree  4. Agree  5. Completely agree | Everyone |
| 36. Der bør ikke være begrænsninger på brug af dyr til medicinsk forskning. | Enkelt svarmulighed  1. Helt uenig  2. Uenig  3. Hverken enig eller uenig  4. Enig  5. Helt enig | Alle |
| 37. Showing dressed, wild animals in circuses is completely acceptable. | Single Response  1. Completely disagree  2. Disagree  3. Neither agree nor disagree  4. Agree  5. Completely agree | Everyone |
| 37. Det er helt acceptabelt at fremvise dresserede vilde dyr i cirkus. | Enkelt svarmulighed  1. Helt uenig  2. Uenig  3. Hverken enig eller uenig  4. Enig  5. Helt enig | Alle |
| 38. Companion animals are entitled to special considerations because they are our friends. | Single Response  1. Completely disagree  2. Disagree  3. Neither agree nor disagree  4. Agree  5. Completely agree | Everyone |
| 38. Kæledyr har krav på særlige hensyn, fordi de er vores venner. | Enkelt svarmulighed  1. Helt uenig  2. Uenig  3. Hverken enig eller uenig  4. Enig  5. Helt enig | Alle |
| 39. Animal welfare is not important when it comes to pests. | Single Response  1. Completely disagree  2. Disagree  3. Neither agree nor disagree  4. Agree  5. Completely agree | Everyone |
| 39. Dyrevelfærd er ikke vigtigt, når det gælder skadedyr. | Enkelt svarmulighed  1. Helt uenig  2. Uenig  3. Hverken enig eller uenig  4. Enig  5. Helt enig | Alle |
| 40. We do not have duties to pests. | Single Response  1. Completely disagree  2. Disagree  3. Neither agree nor disagree  4. Agree  5. Completely agree | Everyone |
| 40. Vi har ikke pligter overfor skadedyr. | Enkelt svarmulighed  1. Helt uenig  2. Uenig  3. Hverken enig eller uenig  4. Enig  5. Helt enig | Alle |
| 41. We have special duties to whales and apes. | Single Response  1. Completely disagree  2. Disagree  3. Neither agree nor disagree  4. Agree  5. Completely agree | Everyone |
| 41. Vi har særlige pligter overfor hvaler og menneskeaber. | Enkelt svarmulighed  1. Helt uenig  2. Uenig  3. Hverken enig eller uenig  4. Enig  5. Helt enig | Alle |
| 42. Our moral duty to animals depends on whether they are animals we have taken in. | Single Response  1. Completely disagree  2. Disagree  3. Neither agree nor disagree  4. Agree  5. Completely agree | Everyone |
| 42. Vores moralske pligt overfor dyr afhænger af, om der er tale om dyr, som vi har taget til os. | Enkelt svarmulighed  1. Helt uenig  2. Uenig  3. Hverken enig eller uenig  4. Enig  5. Helt enig | Alle |
| 43. We have special duties to farm animals because we have taken them in. | Single Response  1. Completely disagree  2. Disagree  3. Neither agree nor disagree  4. Agree  5. Completely agree | Everyone |
| 43. Vi har særlige pligter over for landbrugsdyr, fordi vi har taget dem til os. | Enkelt svarmulighed  1. Helt uenig  2. Uenig  3. Hverken enig eller uenig  4. Enig  5. Helt enig | Alle |
| 44. Our moral duty to animals depends on the role they play in our lives. | Single Response  1. Completely disagree  2. Disagree  3. Neither agree nor disagree  4. Agree  5. Completely agree | Everyone |
| 44. Vores moralske pligt overfor dyr afhænger af, hvilken rolle dyrene spiller i vores liv. | Enkelt svarmulighed  1. Helt uenig  2. Uenig  3. Hverken enig eller uenig  4. Enig  5. Helt enig | Alle |
| 45. After completing the veterinary program, what is your dream job? (you may choose two response options) | Multiple Response (choose two from the list)  1. Practicing veterinarian (small animal practice)  2. Practicing veterinarian (horses)  3. Practicing veterinarian (farm animals)  4. Animal testing  5. Animal research  6. Food safety  7. Not sure  8. Other | Everyone |
| 45. Når du har færdiggjort din uddannelse til dyrlæge, hvad er så dit drømmejob? (du må gerne sætte to krydser) | Flere svarmuligheder (der kan vælges to fra listen)  1. Praktiserende dyrlæge (familiedyr)  2. Praktiserende dyrlæge (heste)  3. Praktiserende dyrlæge (produktionsdyr)  4. Dyreforsøgsforskning  5. Forskning  6. Hygiejne/fødevaresikkerhed  7. Er ikke sikker  8. Andet | Alle |
| 46. How would you categorize yourself? | Single Response  1. I consider myself a meat-eater  2. I consider myself a vegetarian  3. I consider myself a vegan | Everyone |
| 46. Hvordan vil du klassificere dig selv? | Enkelt svarmulighed  1. Jeg anser mig selv for at være kødspiser  2. Jeg anser mig selv for at være vegetar  3. Jeg anser mig selv for at være veganer | Alle |
| 47. How much do you agree with the following statement?  a) I try to eat as little meat as possible. | Single Response  1. To a very great extend  2. To a great extend  3. To some extend  3. Not at all  4. Not relevant (I don’t eat meat) | Everyone |
| 47. Hvor godt passer det følgende udsagn på dig?  a) Jeg bestræber mig på at spise så få kødprodukter som muligt. | Enkelt svarmulighed  1. Passer særdeles godt  2. Passer godt  3. Passer i nogen grad  3. Passer slet ikke  4. Ikke relevant (jeg spiser ikke kød) | Alle |
| 48. How much do you agree with the following statement?  a) I try to eat as few dairy products as possible. | Single Response  1. To a very great extend  2. To a great extend  3. To some extend  3. Not at all  4. Not relevant (I don’t eat dairy products) | Everyone |
| 48. Hvor godt passer det følgende udsagn på dig?  a) Jeg bestræber mig på at spise så få mejeriprodukter som muligt. | Enkelt svarmulighed  1. Passer særdeles godt  2. Passer godt  3. Passer i nogen grad  3. Passer slet ikke  4. Ikke relevant (jeg spiser ikke mejeriprodukter) | Alle |
| 49. How much do you agree with the following statements?  a) I prefer to eat organic food.  b) I am happy to pay a premium price for meat products that have been produced with high levels of animal welfare.  c) I am happy to pay a premium price for dairy products that have been produced with high levels of animal welfare.  d) I avoid eggs from battery-hens.  e) I have no problem buying and wearing fur.  f) I am happy to buy clothes, shoes and the like, made out of leather and other products from animals that are used for agricultural production.  g) I only use cosmetics that are not tested on animals.  h) On principle, I do not visit zoos because the animals are held in captivity.  i) On trips abroad, I avoid activities where live animals are involved (e.g. elephant riding).  j) On trips abroad, I avoid activities involving animals that are not treated well. | Single Response  1. Completely disagree  2. Disagree  3. Neither agree nor disagree  4. Agree  5. Completely agree | Everyone |
| 49. I hvor stort omfang er du enig i nedenstående udsagn?  a) Jeg foretrækker at spise økologisk mad.  b) Jeg betaler gerne ekstra for kødprodukter, der er blevet produceret med ekstra hensyn til dyrevelfærd.  c) Jeg betaler gerne ekstra for mejeriprodukter, der er blevet produceret med ekstra hensyn til dyrevelfærd.  d) Jeg undgår buræg.  e) Jeg har intet problem med at købe og gå med pels.  f) Jeg køber gerne tøj, sko og lignende, fremstillet af læder og andre produkter fra dyr, som i forvejen bruges til landbrugsproduktion.  g) Jeg bruger kun kosmetikprodukter, som ikke er testet på dyr.  h) Jeg går af princip ikke i zoologiske haver, fordi dyrene holdes indespærret.  i) På udlandsrejser undgår jeg aktiviteter, hvor levende dyr indgår (f.eks. elefantridning).  j) På udlandsrejser undgår jeg aktiviteter, hvor der indgår dyr, som ikke bliver behandlet ordentligt. | Enkelt svarmulighed  1. Helt uenig  2. Uenig  3. Hverken enig eller uenig  4. Enig  5. Helt enig | Alle |
