# Supplementary material for: A multidimensional measure of animal ethics orientation – Developed and applied to a representative sample of the Danish public
Source: PLoS One. 2019 Feb 7;14(2):e0211656. doi: 10.1371/journal.pone.0211656 (PMC6366885; doi:10.1371/journal.pone.0211656)
Supplement: S6 Appendix — (DOCX) [file pone.0211656.s024.docx]

| Question / spørgsmål | Response category / svarmulighed | Filter / filter |
| --- | --- | --- |
| 1. Thank you for participating in this survey. The questionnaire is designed by researchers at the Department of Food and Resource Economics. First, we have a few questions about your background.  Do you work within one or more of these job categories? | Multiple responses  1. Farmer or farm assistant  2. Consultant/advisor/vet in livestock productions, slaughterhouses or meat packing companies  3. Strategic, economic or political work concerning livestock productions  4. Butcher, slaughterhouse worker or livestock haulier  5. Work in a meat packing company, meat retail store/butcher's shop  6. No, none of these |  |
| 1. Tak for at du vil medvirke i denne undersøgelse, som udføres af forskere på Københavns Universitet. Først har vi et spørgsmål om dit nuværende job.  Arbejder du indenfor en eller flere af disse jobgrupper? | Flere svarmuligheder  1. Landmand eller landmandsmedhjælper  2. Konsulent / rådgiver / dyrlæge indenfor husdyrproduktion, slagteri, kødforarbejdningsvirksomheder  3. Strategisk, økonomisk eller politisk arbejde omhandlende husdyrproduktion  4. Slagter, slagteriarbejder eller transport af slagtedyr til slagterier  5. Arbejder på en kødforarbejdningsvirksomhed, slagterbutik eller slagteriafdeling  6. Nej, ingen af disse |  |
| 2. Would you describe yourself as: | Single Response  1. Vegetarian: somebody who doesn’t eat meat, poultry, fish or seafood  2. Vegan: somebody who doesn’t eat meat, poultry, fish, seafood, dairy products or eggs  3. One who generally eats vegetarian food but occasionally, i.e. a maximum of 1-2 times a week or on special occasions, eat meat, poultry, fish or seafood  4. None of these |  |
| 2. Vil du betegne dig selv som: | Enkelt svarmulighed  1. Vegetar, altså én der ikke spiser kød, fjerkræ, fisk eller skaldyr  2. Veganer, altså én der ikke spiser kød, fjerkræ, fisk, skaldyr, mejeriprodukter eller æg  3. En, der generelt spiser vegetarisk, men lejlighedsvist, dvs. højest 1-2 gange om ugen eller ved særlige begivenheder, spiser kød, fjerkræ, fisk eller skaldyr  4. Ingen af disse |  |
| 3. Are you: | Single Response  1. Man  2. Woman | Everyone |
| 3. Er du: | Enkelt svarmulighed  1. Mand  2. Kvinde | Alle |
| 4. In what region do you live? | Single Response (the Danish names are used here)  1. Region Hovedstaden  2. Region Sjælland  3. Region Syddanmark  4. Region Midtjylland  5. Abroad  6. I don’t know | Everyone |
| 4. Hvilken region er du bosat i? | Enkelt svarmulighed  1. Region Hovedstaden  2. Region Sjælland  3. Region Syddanmark  4. Region Midtjylland  5. Udlandet  6. Ved ikke | Alle |
| 5. What is your age? (please choose the age group to which you belong) | Single response  1. 15-29 years old  2. 30-34 years old  3. 35-39 years old  4. 40-44 years old  5. 45-49 years old  6. 50-54 years old  7. 55-59 years old  8. 60-64 years old  9. 65-69 years old  10. 70-74 years old  11. 75-79 years old  12. 80 years old or older | Everyone |
| 5. Hvad er din alder? (sæt kryds i den aldersgruppe, der passer på dig) | Enkelt svarmulighed  1. 15-29 år  2. 30-34 år  3. 35-39 år  4. 40-44 år  5. 45-49 år  6. 50-54 år  7. 55-59 år  8. 60-64 år  9. 65-69 år  10. 70-74 år  11. 75-79 år  12. 80 år eller ældre | Alle |
| 6. What is your ZIP code? (please write the digits here) | Open-ended Response  1. __________________ | Everyone |
| 6. Hvad er dit postnummer? (indsæt fire cifret postnummer, fx. 4672) | Åben svarmulighed  1. __________________ | Alle |
| 7. What is your highest level of education obtained? | Single Response  1. Compulsory school  2. High school or equivalent  3. Vocational education  4. Short tertiary education (≤2 years)  5. Medium length tertiary education (2-4½ years)  6. Long tertiary education (≥5 years)  7. Doctorate  8. Other/I don’t know | Everyone |
| 7. Angiv venligst din senest afsluttede uddannelse. | Single Response  1. Grundskole 8.-10. klasse (inkl. realskole)  2. Almengymnasial uddannelse (inkl. HF)  3. Erhvervsgymnasial uddannelse (inkl. HHX og HTX)  4. Erhvervsfaglig uddannelse (fx EUD, håndværksuddannelse, kontoruddannelse, butiksuddannelse) Videregående uddannelse (op til 2 år - Kræver forudgående gymnasial eller erhvervsgymnasial uddannelse) Videregående uddannelse (2-4½ år)  5. Videregående uddannelse (5 år eller længere)  6. Forskeruddannelse (f.eks. Ph.d.) Andet/Ved ikke  7. Andet | Alle |
| 8. On the following pages you will be presented with a number of statements that express attitudes to the use of animals. Please rate the extent to which you agree or disagree with the statements. You may use the following scale going from "completely disagree" to "completely agree". When “animals” and “the use of animals” are mentioned in the statements, please think of animals used for human purposes, e.g. in agricultural production, in animal experiments, or in circuses or zoos.  a) The use of animals by humans should be prohibited by law.  b) In principle, the use of animals by humans is unacceptable because animals can feel pain, happiness, etc.  c) In principle, the use of animals by humans is unacceptable because animals are sentient beings.  d) It is acceptable for humans to put animals down if it is done painlessly.  e) Using animals for important human purposes (e.g. medical research) is acceptable if it is done so that the animals do not experience unnecessary stress.  f) Using animals for important human purposes is acceptable if it is done so that the animals do not experience unnecessary pain and suffering.  g) Using animals for important human purposes is acceptable if the animals have a decent quality of life. | Single Response  1. Completely disagree  2. Disagree  3. Neither agree nor disagree  4. Agree  5. Completely agree | Everyone |
| 8. På de næste sider følger der en række udsagn, som udtrykker holdninger til brug af dyr. Du bedes tage stilling til, hvor enig eller uenig du er i udsagnene. Du kan svare på en skala der går fra ”helt uenig” til ”helt enig”. Når dyr og brug af dyr bliver nævnt i spørgsmålene, bedes du tænke på dyr som anvendes til menneskelige formål, f.eks. til landbrugsproduktion, til dyreforsøg, eller som bliver vist frem i cirkus eller i zoologiske haver.  a) Det burde forbydes ved lov, at mennesker bruger dyr.  b) Det er som udgangspunkt uacceptabelt at mennesker bruger dyr, fordi dyr kan føle smerte, glæde og lignende.  c) Det er som udgangspunkt uacceptabelt at mennesker bruger dyr, fordi dyr er væsner, som sanser og tænker.  d) Det er acceptabelt for mennesker at aflive dyr, hvis det foregår på en måde, så de ikke oplever smerte.  e) Det er acceptabelt at bruge dyr til vigtige menneskelige formål (f.eks. medicinsk forskning), hvis det sikres at dyrene ikke oplever unødig stress.  f) Det er acceptabelt at bruge dyr til vigtige menneskelige formål, hvis det sikres at dyrene ikke oplever unødig smerte og anden lidelse.  g) Det er acceptabelt at bruge dyr til vigtige menneskelige formål, hvis dyrene har et ordentligt liv. | Enkelt svarmulighed  1. Helt uenig  2. Uenig  3. Hverken enig eller uenig  4. Enig  5. Helt enig | Alle |
| 9. Here are some more statements that you are asked to consider.  a) Exposing animals to stress and reducing their welfare is justified if the purpose is sufficiently important.  b) Inflicting considerable pain on animals is justified if the purpose is sufficiently important - e.g. medical research.  c) Inflicting serious pain on animals is acceptable if it is necessary in order to achieve a vital human goal – e.g. in medical research.  d) We have the right to use animals because humans are intellectually superior to animals.  e) We have the right to use animals regardless of the consequences for the animals.  f) Human interests are more important than those of animals.  g) We must prioritize humans over animals. | Single Response  1. Completely disagree  2. Disagree  3. Neither agree nor disagree  4. Agree  5. Completely agree | Everyone |
| 9. Her er nogle flere udsagn, hvor du stadig bedes tage stilling til, hvor enig eller uenig du er.  a) Det kan godt forsvares at dyr oplever stress og velfærdsforringelser, hvis det tjener et formål, som er vigtigt nok.  b) Det kan godt forsvares at påføre dyr væsentlig smerte, hvis formålet er vigtigt nok (f.eks. medicinsk forskning).  c) Det kan være acceptabelt at påføre dyr alvorlig smerte, hvis det er nødvendigt for at opnå et vitalt menneskeligt formål (f.eks. medicinsk forskning).  d) Vi har ret til at bruge dyr, fordi mennesker er intellektuelt overlegne i forhold til dyrene.  e) Vi har ret til at bruge dyr, uanset konsekvenserne for dyrene.  f) Menneskehedens interesser er vigtigere end dyrs interesser.  g) Vi skal tage hensyn til mennesker frem for dyr. | Enkelt svarmulighed  1. Helt uenig  2. Uenig  3. Hverken enig eller uenig  4. Enig  5. Helt enig | Alle |
| 10. You are now presented with a number of statements about our commitment to animals in different contexts.  a) Animal welfare is not important when it comes to rats and other pests.  b) We do not have duties to rats and other pests.  c) Our moral duty to animals depends on whether they are animals we have taken in.  d) Our moral duty to animals depends on the role they play in our lives. | Single Response  1. Completely disagree  2. Disagree  3. Neither agree nor disagree  4. Agree  5. Completely agree | Everyone |
| 10. Nu følger en række spørgsmål om vores forpligtelse over for dyr i forskellige sammenhænge.  a) Dyrevelfærd er ikke vigtigt, når det gælder rotter og andre skadedyr.  b) Vi har ikke pligter overfor rotter og andre skadedyr.  c) Vores moralske pligt overfor dyr afhænger af, om der er tale om dyr, som vi har taget til os.  d) Vores moralske pligt overfor dyr afhænger af, hvilken rolle dyrene spiller i vores liv. | Enkelt svarmulighed  1. Helt uenig  2. Uenig  3. Hverken enig eller uenig  4. Enig  5. Helt enig | Alle |
| 11. To what extend do you agree with the following two statements about animal welfare?  a) All the talk about animal welfare is, in my view, excessive.  b) It has become too trendy to focus on animal welfare. | Single Response  1. Completely disagree  2. Disagree  3. Neither agree nor disagree  4. Agree  5. Completely agree | Everyone |
| 11. Hvor enig er du i de to følgende udsagn om dyrevelfærd?  a) Al den snak om dyrevelfærd er i mine øjne overdreven.  b) Der er gået alt for meget mode i at fokusere på dyrevelfærd. | Enkelt svarmulighed  1. Helt uenig  2. Uenig  3. Hverken enig eller uenig  4. Enig  5. Helt enig | Alle |
| 12. To what extend do you agree with the following statements about animals in zoos?  a) On principle I do not go to zoos, because animals are kept solely for exhibition purposes.  b) To me, it is completely fine to keep animals in zoos as long as they thrive in captivity.  c) Euthanasia of surplus animals in zoos is acceptable if it serves to maintain the welfare of the remaining animals. | Single Response  1. Completely disagree  2. Disagree  3. Neither agree nor disagree  4. Agree  5. Completely agree | Everyone |
| 12. Hvor enig er du i de følgende udsagn om dyr i zoologisk have?  a) Jeg går af princip ikke i zoologiske haver, fordi man der holder dyr med det ene formål at vise dem frem.  b) For mig er det helt fint at holde dyr i zoologiske haver, så længe de trives med at leve i fangenskab.  c) Det er acceptabelt at aflive overskudsdyr i zoologiske haver, hvis det tjener til at sikre velfærden hos de resterende dyr. | Enkelt svarmulighed  1. Helt uenig  2. Uenig  3. Hverken enig eller uenig  4. Enig  5. Helt enig | Alle |
| 13. Here you are presented with a number of statements about the use of animals for various other purposes. Please rate the extent to which you agree or disagree with the statements.  a) Showing dressed, wild animals in circuses is completely acceptable.  b) I have no problem buying and wearing fur.  c) There is no reason to punish persons who have sexual intercourse with animals as long as the animal is not exposed to pain or other discomforts.  d) There should be no restrictions on the use of animals for medical research. | Single Response  1. Completely disagree  2. Disagree  3. Neither agree nor disagree  4. Agree  5. Completely agree | Everyone |
| 13. Her følger en række udsagn, om brug af dyr til forskellige andre formål. Du bedes fortsat tage stilling til, hvor enig eller uenig du er i udsagnene.  a) Det er helt acceptabelt at fremvise dresserede vilde dyr i cirkus.  b) Jeg har intet problem med at købe og gå med pels.  c) Der er ingen grund til at straffe mennesker, der har seksuel omgang med dyr, hvis dyret ikke bliver udsat for smerte eller andet ubehag.  d) Der bør ikke være begrænsninger på brug af dyr til medicinsk forskning. | Enkelt svarmulighed  1. Helt uenig  2. Uenig  3. Hverken enig eller uenig  4. Enig  5. Helt enig | Alle |
| 14. Here you are presented with a number of statements about the use of animals in agricultural production. Please rate the extent to which you agree or disagree with the statements.  a) I am happy to pay a premium price for meat products that have been produced with high levels of animal welfare.  b) I prefer to eat organic food.  c) I avoid eggs from battery-hens.  d) The current legislation is sufficient to ensure that the animals used for meat and dairy production live a decent life.  e) The current principles in organic agriculture are sufficient to ensur that the animals used for meat and dairy production live a decent life. | Single Response  1. Completely disagree  2. Disagree  3. Neither agree nor disagree  4. Agree  5. Completely agree | Everyone |
| 14. Her følger en række spørgsmål om brug af dyr i landbrugsproduktion. Du bedes svare hvor enig eller uenig du er i dem.  a) Jeg betaler gerne ekstra for kødprodukter, der er blevet produceret med ekstra hensyn til dyrevelfærd.  b) Jeg foretrækker at spise økologisk mad.  c) Jeg undgår buræg.  d) Den eksisterende lovgivning er tilstrækkelig til at sikre, at dyrene der bruges i landbruget til produktion af kød og mejeriprodukter, har et ordentligt liv.  e) De eksisterende principper i økologisk landbrug er tilstrækkelige til at sikre, at dyrene der bruges til produktion af kød og mejeriprodukter, har et ordentligt liv. | Enkelt svarmulighed  1. Helt uenig  2. Uenig  3. Hverken enig eller uenig  4. Enig  5. Helt enig | Alle |
| 15. A lot of resources are spent on keeping stray cats in shelters. For a large part of the cats, it is either impossible or very difficult to find a home. What do you think is the right thing to do? | Single Response  1. To euthanise the stray cats that do not find a new home after a short period of time. This saves important resources that could be better spent elsewhere.  2. To be more patient and only consider euthanasia if proper cat welfare is not possible.  3. I have no opinion on this.  4. Other_______________ | Everyone |
| 15. Der bliver brugt rigtig mange ressourcer på at opbevare herreløse katte i internater. For en stor del af kattene er det enten umuligt eller meget svært at finde et hjem. Hvad synes du, er det mest rigtige at gøre? | Enkelt svarmulighed  1. At aflive de herreløse katte, som ikke hurtigt finder et nyt hjem. Hermed spares der vigtige ressourcer, som kan bruges bedre andre steder.  2. At være mere tålmodig og først overveje at aflive, hvis det ikke er muligt at sikre en forsvarlig velfærd for katten.  3. Jeg har ikke nogen mening om dette.  4. Andet_______________ | Alle |
| 16. You are now presented with a number of statements about your perception of meat. Please use the following scale going from "very strongly disagree" to "very strongly agree".  a) It is only natural to eat meat.  b) It is unnatural to eat an all plant-based diet.  c) Our human ancestors ate meat all the time.  d) Human beings naturally crave meat  e) It is necessary to eat meat in order to be healthy.  f) You cannot get all the protein, vitamins, and mineral you need on an all plant-based diet.  g) Human beings need to eat meat.  h) A healthy diet requires at least some meat. | Single Response  1. Completely disagree  2. Disagree  3. Slightly disagree  4. Neither agree nor disagree  5. Slightly agree  6. Agree  7. Completely agree | Everyone |
| 16. Nu følger nogle spørgsmål, om din opfattelse af kød. Du bedes svare på en skala, der går fra ”Ekstremt uenig” til ”Ekstremt enig”.  a) Det er helt naturligt, at spise kød.  b) Det er unaturligt, at spise en kost, som udelukkende består af planter.  c) Vores forfædre spiste kød hele tiden.  d) Det er naturligt for mennesker, at have en stærk lyst til at spise kød.  e) Det er nødvendigt, at spise kød for at være sund.  f) Man får ikke de nødvendige proteiner, vitaminer og mineraler, hvis man spiser en kost, der kun er baseret på planter.  g) Mennesker har brug for at spise kød.  h) En sund kost kræver som mindstemål en vis mængde kød. | Enkelt svarmulighed  1. Ekstremt uenig  2. Helt uenig  3. Delvist uenig  4. Hverken enig eller uenig  5. Delvist enig  6. Helt enig  7. Ekstremt enig | Alle |
| 17. Finally, a few more questions about your perception of meat.  a) Not eating meat is socially unacceptable.  b) It is abnormal for humans not to eat meat.  c) Most people I know eat meat.  d) It is normal to eat meat.  e) Meat is delicious.  f) Meat adds so much flavour to a meal it does not make sense to leave it out.  g) The best tasting food is normally a meat-based dish (e.g., steak, chicken breast, grilled fish).  h) Meals without meat would just be bland and boring. | Single Response  1. Completely disagree  2. Disagree  3. Slightly disagree  4. Neither agree nor disagree  5. Slightly agree  6. Agree  7. Completely agree | Everyone |
| 17. Til sidst følger lidt flere spørgsmål om din opfattelse af kød.  a) Det er socialt uacceptabelt ikke at spise kød.  b) Det er afvigende, når mennesker ikke spiser kød.  c) De fleste mennesker, jeg kender, spiser kød.  d) Det er normalt at spise kød.  e) Kød er lækkert.  f) Kød giver så meget smag til et måltid, at det ikke giver mening at udelade det.  g) Den mest velsmagende mad er normalt retter baseret på kød (f.eks. bøf, kyllingebryst, grillet fisk).  h) Måltider uden kød ville være uinteressante og kedelige. | Enkelt svarmulighed  1. Ekstremt uenig  2. Helt uenig  3. Delvist uenig  4. Hverken enig eller uenig  5. Delvist enig  6. Helt enig  7. Ekstremt enig | Alle |
