# Supplementary material for: A multidimensional measure of animal ethics orientation – Developed and applied to a representative sample of the Danish public
Source: PLoS One. 2019 Feb 7;14(2):e0211656. doi: 10.1371/journal.pone.0211656 (PMC6366885; doi:10.1371/journal.pone.0211656)
Supplement: S7 Appendix — (DOCX) [file pone.0211656.s025.docx]

Thank you for participating in this survey. The questionnaire is designed by researchers at the Department of Food and Resource Economics. The results from the survey will be part of a research project about attitudes to farm animal welfare and to animal use in various other contexts.

First, we have a few questions about your background.

**Q1 What is your gender?**

1. Man

2. Woman

**Q2 How is your household composed?**

1. I live with my parents

2. I live alone

3. I live with my kid/s

4. I live with my partner without children at home

5. I live with my partner and children at home

6. Other, e.g. house share or college

**Q3 What is your date of birth?**

Numerical (between 1942-1999)

**Q4 What is your ZIP code?**

Numerical (between 1000-9999)

**Q5 What is your highest level of education obtained?**

1. Compulsory school

2. High school or equivalent

3. Vocational education

4. Short tertiary education (≤2 years)

5. Medium length tertiary education (2-4½ years)

6. Long tertiary education (≥5 years)

7. Doctorate

8. Other/I don’t know

**Q6 What is your current employment status**

1. Self-employed

2. Employed (publicly or privately)

3. On leave

4. Unemployed (currently looking for work)

5. Unemployed (currently not looking for work)

6. Retired

7. Homemaker

8. Student

9. Other

On the following pages you will be presented with a number of statements that express attitudes to the use of animals. Please rate the extent to which you agree or disagree with the statements. You may use the following scale going from "completely disagree" to "completely agree". When “animals” and “the use of animals” are mentioned in the statements, please think of animals used for human purposes, e.g. in agricultural production, in animal experiments, or in circuses or zoos.

(Randomised)

**Q7 The use of animals by humans should be prohibited by law.**

**Q8 In principle, the use of animals by humans is unacceptable because animals can feel pain, happiness, etc.**

**Q9 In principle, the use of animals by humans is unacceptable because animals are sentient beings.**

**Q10 It is acceptable for humans to put animals down if it is done painlessly.**

**Q11 Using animals for important human purposes (e.g. medical research) is acceptable if it is done so that the animals do not experience unnecessary stress.**

**Q12 Using animals for important human purposes is acceptable if it is done so that the animals do not experience unnecessary pain and suffering.**

**Q13 Using animals for important human purposes is acceptable if the animals have a decent quality of life.**

1. Completely agree

2. Agree

3. Neither agree nor disagree

4. Disagree

5. Completely disagree

Here are some more statements that you are asked to consider.

(Randomised)

**Q14 Exposing animals to stress and reducing their welfare is justified if the purpose is sufficiently important.**

**Q15 Inflicting considerable pain on animals is justified if the purpose is sufficiently important - e.g. medical research.**

**Q16 Inflicting serious pain on animals is acceptable if it is necessary in order to achieve a vital human goal – e.g. in medical research.**

**Q17 We have the right to use animals because humans are intellectually superior to animals.**

**Q18 We have the right to use animals regardless of the consequences for the animals.**

**Q19 Human interests are more important than those of animals.**

**Q20 We must prioritize humans over animals.**

1. Completely agree

2. Agree

3. Neither agree nor disagree

4. Disagree

5. Completely disagree

You are now presented with a number of statements about our commitment to animals in different contexts.

(Randomised)

**Q21 Animal welfare is not important when it comes to rats and other pests.**

**Q22 We do not have duties to rats and other pests.**

1. Completely agree

2. Agree

3. Neither agree nor disagree

4. Disagree

5. Completely disagree

To what extend do you agree with the following statements about animal welfare?

(Randomised)

**Q23 All the talk about animal welfare is, in my view, excessive.**

**Q24 It has become too trendy to focus on animal welfare.**

**Q25 There are more urgent matters for society to deal with than the living conditions for animals.**

**Q26 The current legislation is sufficient to ensure that the animals used for meat and dairy production live a decent life.**

1. Completely agree

2. Agree

3. Neither agree nor disagree

4. Disagree

5. Completely disagree

(A four-block split is created (Block A to D) and respondents are randomised to one of the blocks)

**Block A questions**

Now, we would like to ask you about your perception of the messages in the following two ads that were used in recent campaign. You can see the first ad here.

(In the table below, the original Danish poster is shown in the left-hand column, while an English translation is provided in the right-hand column.)

| **ORIGINAL POSTER (presented to respondents)** | **ENGLISH TRANSLATION** |
| --- | --- |
| 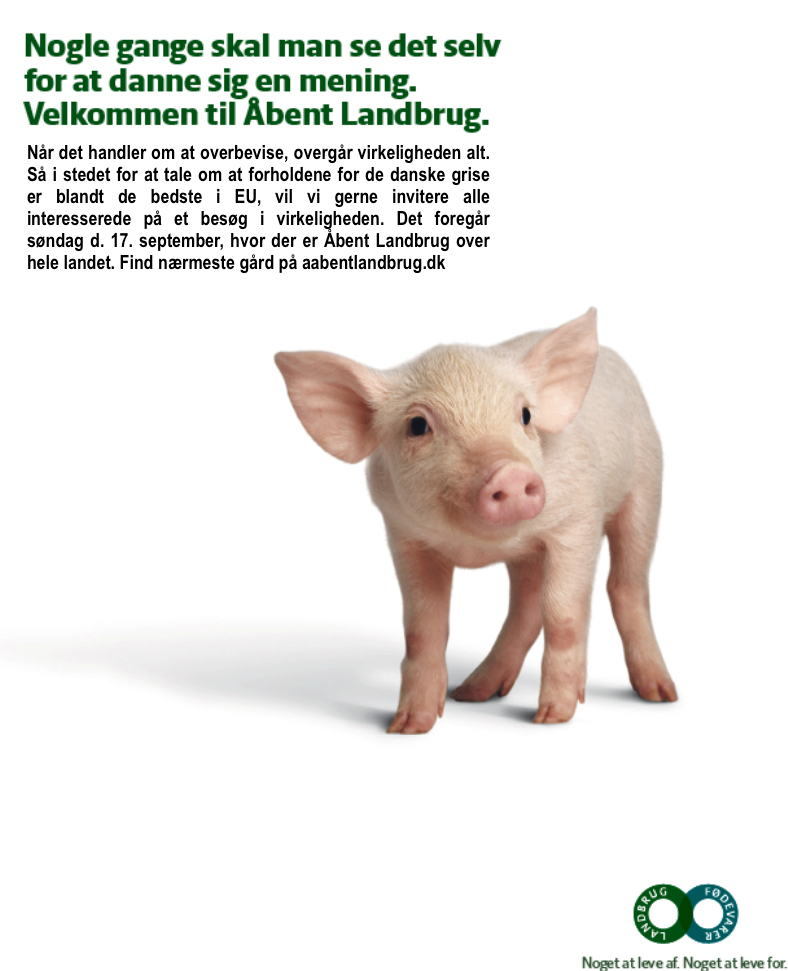 | **Sometimes you have to see it yourself to form an attitude. Welcome to Open Farm.**  When it comes to persuading, reality trumps everything else. So instead of just telling you that the conditions for pigs in Denmark are among the best in the EU, we would like to invite you to a visit into reality. Throughout the country, we have Open Farm on Sunday 17^th^ September. Find your nearest farm on aabentlandbrug.dk.  **Farm and Food** |

**Q27a First we would like to know if the add was displayed on your screen (a picture and a text)?**

1. Yes

2. No (jump to Q33a)

**Q28a Have you seen this ad before, in newspapers, magazines, or on billboards on the street?**

1. Yes, definitely

2. Yes, I believe so

3. No, I don’t remember

4. No, definitely not

5. I’m not sure

**To which extend do you agree or disagree with the following statements about the campaign?**

**Q29a I have great sympathy for the campaign**

**Q30a The message in the campaign is misleading**

**Q31a The campaign is good because it says something central about the way pigs are treated in Danish farms**

1. Completely agree

2. Agree

3. Neither agree nor disagree

4. Disagree

5. Completely disagree

(Q32a is only presented if Q31a = ‘Completely agree’ or ‘Agree’)

**Q32a In your view, what does the campaign say about pig farming in Denmark?**

(Open-ended question)

Here is the next ad.

(In the table below, the original Danish poster is shown in the left-hand column, while an English translation is provided in the right-hand column.)

| **ORIGINAL POSTER (presented to respondents)** | **ENGLISH TRANSLATION** |
| --- | --- |
|  | **Name of the NGO**  **(Dyrenes Beskyttelse)**  **IF ONLY PIGS WERE HENS**  You have said no to battery hens – now the pig needs your help  BATTERY **NO**  PIGS **THANKS** |

**Q33a First, we would like to know if the add was displayed on your screen (a picture and a text)?**

1. Yes

2. No (jump to vignette questions under ‘Pig welfare in agriculture’)

**Q34a Have you seen this ad before, in newspapers, magazines, or on billboards on the street?**

1. Yes, definitely

2. Yes, I believe so

3. No, I don’t remember

4. No, definitely not

5. I’m not sure

**To which extend do you agree or disagree with the following statements about the campaign?**

**Q35a I have great sympathy for the campaign**

**Q36a The message in the campaign is misleading**

**Q37a The campaign is good because it says something central about the way pigs are treated in Danish farms**

1. Completely agree

2. Agree

3. Neither agree nor disagree

4. Disagree

5. Completely disagree

(Q38a is only presented if Q37a = ‘Completely agree’ or ‘Agree’)

**Q38a In your view, what does the campaign say about pig farming in Denmark?**

(Open-ended question)

**Block B questions**

Now, we would like to ask you about your perception of the messages in the following two ads that were used in recent campaign. You can see the first ad here.

(In the table below, the original Danish poster is shown in the left-hand column, while an English translation is provided in the right-hand column.)

| **ORIGINAL POSTER (presented to respondents)** | **ENGLISH TRANSLATION** |
| --- | --- |
|  | **Name of the NGO**  **(Dyrenes Beskyttelse)**  **IF ONLY PIGS WERE HENS**  You have said no to battery hens – now the pig needs your help  BATTERY **NO**  PIGS **THANKS** |

**Q27b First, we would like to know if the add was displayed on your screen (a picture and a text)?**

1. Yes

2. No (jump to 33b)

**Q28b Have you seen this ad before, in newspapers, magazines, or on billboards on the street?**

1. Yes, definitely

2. Yes, I believe so

3. No, I don’t remember

4. No, definitely not

5. I’m not sure

**To which extend do you agree or disagree with the following statements about the campaign?**

**Q29b I have great sympathy for the campaign**

**Q30b The message in the campaign is misleading**

**Q31b The campaign is good because it says something central about the way pigs are treated in Danish farms**

1. Completely agree

2. Agree

3. Neither agree nor disagree

4. Disagree

5. Completely disagree

(Q32b is only presented if Q31b = ‘Completely agree’ or ‘Agree’)

**Q32b In your view, what does the campaign say about pig farming in Denmark?**

(Open-ended question)

Here is the next ad.

(In the table below, the original Danish poster is shown in the left-hand column, while an English translation is provided in the right-hand column.)

| **ORIGINAL POSTER (presented to respondents)** | **ENGLISH TRANSLATION** |
| --- | --- |
| 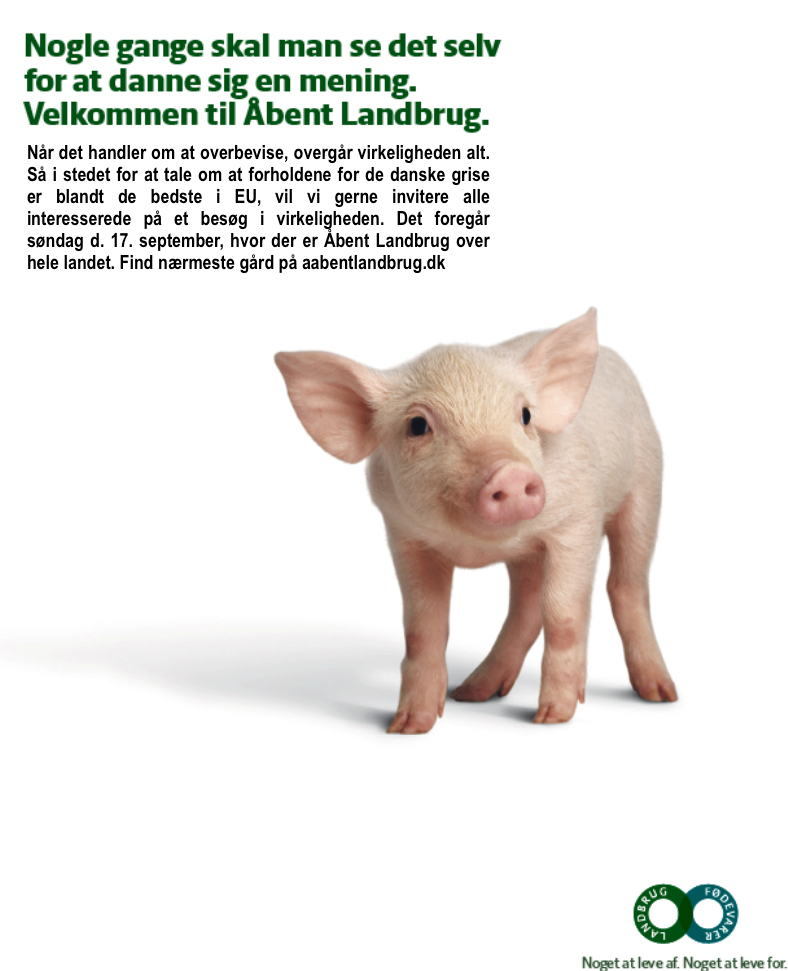 | **Sometimes you have to see it yourself to form an attitude. Welcome to Open Farm.**  When it comes to persuading, reality trumps everything else. So instead of just telling you that the conditions for pigs in Denmark are among the best in the EU, we would like to invite you to a visit into reality. Throughout the country, we have Open Farm on Sunday 17^th^ September. Find your nearest farm on aabentlandbrug.dk.  **Farm and Food** |

**Q33b First we would like to know if the add was displayed on your screen (a picture and a text)?**

1. Yes

2. No (jump to vignette questions under ‘Pig welfare in agriculture’)

**Q34b Have you seen this ad before, in newspapers, magazines, or on billboards on the street?**

1. Yes, definitely

2. Yes, I believe so

3. No, I don’t remember

4. No, definitely not

5. I’m not sure

**To which extend do you agree or disagree with the following statements about the campaign?**

**Q35b I have great sympathy for the campaign**

**Q36b The message in the campaign is misleading**

**Q37b The campaign is good because it says something central about the way pigs are treated in Danish farms**

1. Completely agree

2. Agree

3. Neither agree nor disagree

4. Disagree

5. Completely disagree

(Q38b is only presented if Q37b = ‘Completely agree’ or ‘Agree’)

**Q38b In your view, what does the campaign say about pig farming in Denmark?**

(Open-ended question)

**Pig welfare in agriculture**

The living conditions for pigs can be different depending on the type of livestock production – whether we are dealing with conventional or alternative productions systems. For example, the space allowance per pig and rooting material vary between the systems.

With the next questions, we ask you to evaluate the welfare of pigs in different production systems. The housing conditions and situations for the pigs are different in the presented cases. They differ with regard to the four aspects of pig housing that you will find in the table below.

| **The number of pigs that are underweight:** If there are underweight pigs in a farm it is a sign of starvation, likely due to inadequate feeding conditions.  **The number of pigs that have been exposed to tail biting:** Tail biting in pigs occurs when the housing conditions are not optimal, e.g. temperature variation, lack of rooting materials.  **If there is sufficient space to lie down when resting:** Whether pigs on a farm can easily lay down on one side when resting, without other pigs getting in the way.  **If there are enrichment materials for rooting and playing:** Whether pigs on a farm have access to straw, hay, wood or the like, that pigs root and play with in nature. |
| --- |

Besides the aspects of pig housing displayed in the table, please imagine that the conditions for the pigs and the production systems are similar. Imaging, for example, that the number of pigs on the farms and the geographical locations of the farms are exactly alike.

**How to evaluate the welfare of the pigs**

For all farms you are presented with, please evaluate the pig welfare on a scale going from 0 to 100, where 0 = “Extremely low welfare” and 100 = “Very high welfare”.

You are also asked to evaluate whether the welfare for the pigs is “Very acceptable”, “Acceptable”, “Should be improved”, “So poor that the farm should be monitored”, and “So poor that the farm should be closed”.

(A four-block split is created (Block E to H) and respondents are randomised to one of the blocks)

Block E receives the following vignettes: 4, 7, 10, 13, 18, 23

Block F receives the following vignettes: 5, 6, 9, 16, 19, 20

Block G receives the following vignettes: 2, 3, 12, 15, 21, 22

Block H receives the following vignettes: 1, 8, 11, 14, 17, 24

**Vignette 4**

| Please imagine a pig farm where: |  | |
| --- | --- | --- |
| The proportion of underweight pigs: | | 0% |
| The proportion of pigs affected by tail biting: | | 0% |
| Resting area: Is there sufficient space to lie down on concrete floor when the pigs rest? | | No |
| Enrichment materials: Are there enrichment materials that the pigs can root and play with? | | No |

**How would you characterize the welfare for the pigs on this farm?**

Please rate the welfare on a scale where 0 = “Extremely low welfare” and 100 = “Very high welfare”.

Numerical (from 0 to 100)

**Do you think the welfare for the pigs is…?**

1. Very acceptable

2. Acceptable

3. Should be improved

4. So poor that the farm should be monitored

5. So poor that the farm should be closed

6. I don’t know

**Vignette 7**

| Please imagine a pig farm where: |  | |
| --- | --- | --- |
| The proportion of underweight pigs: | | 0% |
| The proportion of pigs affected by tail biting: | | 15% |
| Resting area: Is there sufficient space to lie down on concrete floor when the pigs rest? | | No |
| Enrichment materials: Are there enrichment materials that the pigs can root and play with? | | Yes |

**How would you characterize the welfare for the pigs on this farm?**

Please rate the welfare on a scale where 0 = “Extremely low welfare” and 100 = “Very high welfare”.

Numerical (from 0 to 100)

**Do you think the welfare for the pigs is…?**

1. Very acceptable

2. Acceptable

3. Should be improved

4. So poor that the farm should be monitored

5. So poor that the farm should be closed

6. I don’t know

**Vignette 10**

| Please imagine a pig farm where: |  | |
| --- | --- | --- |
| The proportion of underweight pigs: | | 1% |
| The proportion of pigs affected by tail biting: | | 0% |
| Resting area: Is there sufficient space to lie down on concrete floor when the pigs rest? | | Yes |
| Enrichment materials: Are there enrichment materials that the pigs can root and play with? | | No |

**How would you characterize the welfare for the pigs on this farm?**

Please rate the welfare on a scale where 0 = “Extremely low welfare” and 100 = “Very high welfare”.

Numerical (from 0 to 100)

**Do you think the welfare for the pigs is…?**

1. Very acceptable

2. Acceptable

3. Should be improved

4. So poor that the farm should be monitored

5. So poor that the farm should be closed

6. I don’t know

**Vignette 13**

| Please imagine a pig farm where: |  | |
| --- | --- | --- |
| The proportion of underweight pigs: | | 1% |
| The proportion of pigs affected by tail biting: | | 15% |
| Resting area: Is there sufficient space to lie down on concrete floor when the pigs rest? | | Yes |
| Enrichment materials: Are there enrichment materials that the pigs can root and play with? | | Yes |

**How would you characterize the welfare for the pigs on this farm?**

Please rate the welfare on a scale where 0 = “Extremely low welfare” and 100 = “Very high welfare”.

Numerical (from 0 to 100)

**Do you think the welfare for the pigs is…?**

1. Very acceptable

2. Acceptable

3. Should be improved

4. So poor that the farm should be monitored

5. So poor that the farm should be closed

6. I don’t know

**Vignette 18**

| Please imagine a pig farm where: |  | |
| --- | --- | --- |
| The proportion of underweight pigs: | | 10% |
| The proportion of pigs affected by tail biting: | | 0% |
| Resting area: Is there sufficient space to lie down on concrete floor when the pigs rest? | | Yes |
| Enrichment materials: Are there enrichment materials that the pigs can root and play with? | | No |

**How would you characterize the welfare for the pigs on this farm?**

Please rate the welfare on a scale where 0 = “Extremely low welfare” and 100 = “Very high welfare”.

Numerical (from 0 to 100)

**Do you think the welfare for the pigs is…?**

1. Very acceptable

2. Acceptable

3. Should be improved

4. So poor that the farm should be monitored

5. So poor that the farm should be closed

6. I don’t know

**Vignette 23**

| Please imagine a pig farm where: |  | |
| --- | --- | --- |
| The proportion of underweight pigs: | | 10% |
| The proportion of pigs affected by tail biting: | | 15% |
| Resting area: Is there sufficient space to lie down on concrete floor when the pigs rest? | | No |
| Enrichment materials: Are there enrichment materials that the pigs can root and play with? | | Yes |

**How would you characterize the welfare for the pigs on this farm?**

Please rate the welfare on a scale where 0 = “Extremely low welfare” and 100 = “Very high welfare”.

Numerical (from 0 to 100)

**Do you think the welfare for the pigs is…?**

1. Very acceptable

2. Acceptable

3. Should be improved

4. So poor that the farm should be monitored

5. So poor that the farm should be closed

6. I don’t know

**Vignette 5**

| Please imagine a pig farm where: |  | |
| --- | --- | --- |
| The proportion of underweight pigs: | | 0% |
| The proportion of pigs affected by tail biting: | | 15% |
| Resting area: Is there sufficient space to lie down on concrete floor when the pigs rest? | | Yes |
| Enrichment materials: Are there enrichment materials that the pigs can root and play with? | | Yes |

**How would you characterize the welfare for the pigs on this farm?**

Please rate the welfare on a scale where 0 = “Extremely low welfare” and 100 = “Very high welfare”.

Numerical (from 0 to 100)

**Do you think the welfare for the pigs is…?**

1. Very acceptable

2. Acceptable

3. Should be improved

4. So poor that the farm should be monitored

5. So poor that the farm should be closed

6. I don’t know

**Vignette 6**

| Please imagine a pig farm where: |  | |
| --- | --- | --- |
| The proportion of underweight pigs: | | 0% |
| The proportion of pigs affected by tail biting: | | 15% |
| Resting area: Is there sufficient space to lie down on concrete floor when the pigs rest? | | Yes |
| Enrichment materials: Are there enrichment materials that the pigs can root and play with? | | No |

**How would you characterize the welfare for the pigs on this farm?**

Please rate the welfare on a scale where 0 = “Extremely low welfare” and 100 = “Very high welfare”.

Numerical (from 0 to 100)

**Do you think the welfare for the pigs is…?**

1. Very acceptable

2. Acceptable

3. Should be improved

4. So poor that the farm should be monitored

5. So poor that the farm should be closed

6. I don’t know

**Vignette 9**

| Please imagine a pig farm where: |  | |
| --- | --- | --- |
| The proportion of underweight pigs: | | 1% |
| The proportion of pigs affected by tail biting: | | 0% |
| Resting area: Is there sufficient space to lie down on concrete floor when the pigs rest? | | Yes |
| Enrichment materials: Are there enrichment materials that the pigs can root and play with? | | Yes |

**How would you characterize the welfare for the pigs on this farm?**

Please rate the welfare on a scale where 0 = “Extremely low welfare” and 100 = “Very high welfare”.

Numerical (from 0 to 100)

**Do you think the welfare for the pigs is…?**

1. Very acceptable

2. Acceptable

3. Should be improved

4. So poor that the farm should be monitored

5. So poor that the farm should be closed

6. I don’t know

**Vignette 16**

| Please imagine a pig farm where: |  | |
| --- | --- | --- |
| The proportion of underweight pigs: | | 1% |
| The proportion of pigs affected by tail biting: | | 15% |
| Resting area: Is there sufficient space to lie down on concrete floor when the pigs rest? | | No |
| Enrichment materials: Are there enrichment materials that the pigs can root and play with? | | No |

**How would you characterize the welfare for the pigs on this farm?**

Please rate the welfare on a scale where 0 = “Extremely low welfare” and 100 = “Very high welfare”.

Numerical (from 0 to 100)

**Do you think the welfare for the pigs is…?**

1. Very acceptable

2. Acceptable

3. Should be improved

4. So poor that the farm should be monitored

5. So poor that the farm should be closed

6. I don’t know

**Vignette 19**

| Please imagine a pig farm where: |  | |
| --- | --- | --- |
| The proportion of underweight pigs: | | 10% |
| The proportion of pigs affected by tail biting: | | 0% |
| Resting area: Is there sufficient space to lie down on concrete floor when the pigs rest? | | No |
| Enrichment materials: Are there enrichment materials that the pigs can root and play with? | | Yes |

**How would you characterize the welfare for the pigs on this farm?**

Please rate the welfare on a scale where 0 = “Extremely low welfare” and 100 = “Very high welfare”.

Numerical (from 0 to 100)

**Do you think the welfare for the pigs is…?**

1. Very acceptable

2. Acceptable

3. Should be improved

4. So poor that the farm should be monitored

5. So poor that the farm should be closed

6. I don’t know

**Vignette 20**

| Please imagine a pig farm where: |  | |
| --- | --- | --- |
| The proportion of underweight pigs: | | 10% |
| The proportion of pigs affected by tail biting: | | 0% |
| Resting area: Is there sufficient space to lie down on concrete floor when the pigs rest? | | No |
| Enrichment materials: Are there enrichment materials that the pigs can root and play with? | | No |

**How would you characterize the welfare for the pigs on this farm?**

Please rate the welfare on a scale where 0 = “Extremely low welfare” and 100 = “Very high welfare”.

Numerical (from 0 to 100)

**Do you think the welfare for the pigs is…?**

1. Very acceptable

2. Acceptable

3. Should be improved

4. So poor that the farm should be monitored

5. So poor that the farm should be closed

6. I don’t know

**Vignette 2**

| Please imagine a pig farm where: |  | |
| --- | --- | --- |
| The proportion of underweight pigs: | | 0% |
| The proportion of pigs affected by tail biting: | | 0% |
| Resting area: Is there sufficient space to lie down on concrete floor when the pigs rest? | | Yes |
| Enrichment materials: Are there enrichment materials that the pigs can root and play with? | | No |

**How would you characterize the welfare for the pigs on this farm?**

Please rate the welfare on a scale where 0 = “Extremely low welfare” and 100 = “Very high welfare”.

Numerical (from 0 to 100)

**Do you think the welfare for the pigs is…?**

1. Very acceptable

2. Acceptable

3. Should be improved

4. So poor that the farm should be monitored

5. So poor that the farm should be closed

6. I don’t know

**Vignette 3**

| Please imagine a pig farm where: |  | |
| --- | --- | --- |
| The proportion of underweight pigs: | | 0% |
| The proportion of pigs affected by tail biting: | | 0% |
| Resting area: Is there sufficient space to lie down on concrete floor when the pigs rest? | | No |
| Enrichment materials: Are there enrichment materials that the pigs can root and play with? | | Yes |

**How would you characterize the welfare for the pigs on this farm?**

Please rate the welfare on a scale where 0 = “Extremely low welfare” and 100 = “Very high welfare”.

Numerical (from 0 to 100)

**Do you think the welfare for the pigs is…?**

1. Very acceptable

2. Acceptable

3. Should be improved

4. So poor that the farm should be monitored

5. So poor that the farm should be closed

6. I don’t know

**Vignette 12**

| Please imagine a pig farm where: |  | |
| --- | --- | --- |
| The proportion of underweight pigs: | | 1% |
| The proportion of pigs affected by tail biting: | | 0% |
| Resting area: Is there sufficient space to lie down on concrete floor when the pigs rest? | | No |
| Enrichment materials: Are there enrichment materials that the pigs can root and play with? | | No |

**How would you characterize the welfare for the pigs on this farm?**

Please rate the welfare on a scale where 0 = “Extremely low welfare” and 100 = “Very high welfare”.

Numerical (from 0 to 100)

**Do you think the welfare for the pigs is…?**

1. Very acceptable

2. Acceptable

3. Should be improved

4. So poor that the farm should be monitored

5. So poor that the farm should be closed

6. I don’t know

**Vignette 15**

| Please imagine a pig farm where: |  | |
| --- | --- | --- |
| The proportion of underweight pigs: | | 1% |
| The proportion of pigs affected by tail biting: | | 15% |
| Resting area: Is there sufficient space to lie down on concrete floor when the pigs rest? | | No |
| Enrichment materials: Are there enrichment materials that the pigs can root and play with? | | Yes |

**How would you characterize the welfare for the pigs on this farm?**

Please rate the welfare on a scale where 0 = “Extremely low welfare” and 100 = “Very high welfare”.

Numerical (from 0 to 100)

**Do you think the welfare for the pigs is…?**

1. Very acceptable

2. Acceptable

3. Should be improved

4. So poor that the farm should be monitored

5. So poor that the farm should be closed

6. I don’t know

**Vignette 21**

| Please imagine a pig farm where: |  | |
| --- | --- | --- |
| The proportion of underweight pigs: | | 10% |
| The proportion of pigs affected by tail biting: | | 15% |
| Resting area: Is there sufficient space to lie down on concrete floor when the pigs rest? | | Yes |
| Enrichment materials: Are there enrichment materials that the pigs can root and play with? | | Yes |

**How would you characterize the welfare for the pigs on this farm?**

Please rate the welfare on a scale where 0 = “Extremely low welfare” and 100 = “Very high welfare”.

Numerical (from 0 to 100)

**Do you think the welfare for the pigs is…?**

1. Very acceptable

2. Acceptable

3. Should be improved

4. So poor that the farm should be monitored

5. So poor that the farm should be closed

6. I don’t know

**Vignette 22**

| Please imagine a pig farm where: |  | |
| --- | --- | --- |
| The proportion of underweight pigs: | | 10% |
| The proportion of pigs affected by tail biting: | | 15% |
| Resting area: Is there sufficient space to lie down on concrete floor when the pigs rest? | | Yes |
| Enrichment materials: Are there enrichment materials that the pigs can root and play with? | | No |

**How would you characterize the welfare for the pigs on this farm?**

Please rate the welfare on a scale where 0 = “Extremely low welfare” and 100 = “Very high welfare”.

Numerical (from 0 to 100)

**Do you think the welfare for the pigs is…?**

1. Very acceptable

2. Acceptable

3. Should be improved

4. So poor that the farm should be monitored

5. So poor that the farm should be closed

6. I don’t know

**Vignette 1**

| Please imagine a pig farm where: |  | |
| --- | --- | --- |
| The proportion of underweight pigs: | | 0% |
| The proportion of pigs affected by tail biting: | | 0% |
| Resting area: Is there sufficient space to lie down on concrete floor when the pigs rest? | | Yes |
| Enrichment materials: Are there enrichment materials that the pigs can root and play with? | | Yes |

**How would you characterize the welfare for the pigs on this farm?**

Please rate the welfare on a scale where 0 = “Extremely low welfare” and 100 = “Very high welfare”.

Numerical (from 0 to 100)

**Do you think the welfare for the pigs is…?**

1. Very acceptable

2. Acceptable

3. Should be improved

4. So poor that the farm should be monitored

5. So poor that the farm should be closed

6. I don’t know

**Vignette 8**

| Please imagine a pig farm where: |  | |
| --- | --- | --- |
| The proportion of underweight pigs: | | 0% |
| The proportion of pigs affected by tail biting: | | 15% |
| Resting area: Is there sufficient space to lie down on concrete floor when the pigs rest? | | No |
| Enrichment materials: Are there enrichment materials that the pigs can root and play with? | | No |

**How would you characterize the welfare for the pigs on this farm?**

Please rate the welfare on a scale where 0 = “Extremely low welfare” and 100 = “Very high welfare”.

Numerical (from 0 to 100)

**Do you think the welfare for the pigs is…?**

1. Very acceptable

2. Acceptable

3. Should be improved

4. So poor that the farm should be monitored

5. So poor that the farm should be closed

6. I don’t know

**Vignette 11**

| Please imagine a pig farm where: |  | |
| --- | --- | --- |
| The proportion of underweight pigs: | | 1% |
| The proportion of pigs affected by tail biting: | | 0% |
| Resting area: Is there sufficient space to lie down on concrete floor when the pigs rest? | | No |
| Enrichment materials: Are there enrichment materials that the pigs can root and play with? | | Yes |

**How would you characterize the welfare for the pigs on this farm?**

Please rate the welfare on a scale where 0 = “Extremely low welfare” and 100 = “Very high welfare”.

Numerical (from 0 to 100)

**Do you think the welfare for the pigs is…?**

1. Very acceptable

2. Acceptable

3. Should be improved

4. So poor that the farm should be monitored

5. So poor that the farm should be closed

6. I don’t know

**Vignette 14**

| Please imagine a pig farm where: |  | |
| --- | --- | --- |
| The proportion of underweight pigs: | | 1% |
| The proportion of pigs affected by tail biting: | | 15% |
| Resting area: Is there sufficient space to lie down on concrete floor when the pigs rest? | | Yes |
| Enrichment materials: Are there enrichment materials that the pigs can root and play with? | | No |

**How would you characterize the welfare for the pigs on this farm?**

Please rate the welfare on a scale where 0 = “Extremely low welfare” and 100 = “Very high welfare”.

Numerical (from 0 to 100)

**Do you think the welfare for the pigs is…?**

1. Very acceptable

2. Acceptable

3. Should be improved

4. So poor that the farm should be monitored

5. So poor that the farm should be closed

6. I don’t know

**Vignette 17**

| Please imagine a pig farm where: |  | |
| --- | --- | --- |
| The proportion of underweight pigs: | | 10% |
| The proportion of pigs affected by tail biting: | | 0% |
| Resting area: Is there sufficient space to lie down on concrete floor when the pigs rest? | | Yes |
| Enrichment materials: Are there enrichment materials that the pigs can root and play with? | | Yes |

**How would you characterize the welfare for the pigs on this farm?**

Please rate the welfare on a scale where 0 = “Extremely low welfare” and 100 = “Very high welfare”.

Numerical (from 0 to 100)

**Do you think the welfare for the pigs is…?**

1. Very acceptable

2. Acceptable

3. Should be improved

4. So poor that the farm should be monitored

5. So poor that the farm should be closed

6. I don’t know

**Vignette 24**

| Please imagine a pig farm where: |  | |
| --- | --- | --- |
| The proportion of underweight pigs: | | 10% |
| The proportion of pigs affected by tail biting: | | 15% |
| Resting area: Is there sufficient space to lie down on concrete floor when the pigs rest? | | No |
| Enrichment materials: Are there enrichment materials that the pigs can root and play with? | | No |

**How would you characterize the welfare for the pigs on this farm?**

Please rate the welfare on a scale where 0 = “Extremely low welfare” and 100 = “Very high welfare”.

Numerical (from 0 to 100)

**Do you think the welfare for the pigs is…?**

1. Very acceptable

2. Acceptable

3. Should be improved

4. So poor that the farm should be monitored

5. So poor that the farm should be closed

6. I don’t know

**Block C questions**

Now, we would like to ask you about your perception of the messages in the following two ads that were used in recent campaign. You can see the first ad here.

(In the table below, the original Danish poster is shown in the left-hand column, while an English translation is provided in the right-hand column.)

| **ORIGINAL POSTER (presented to respondents)** | **ENGLISH TRANSLATION** |
| --- | --- |
|  | **Name of the NGO**  **(Dyrenes Beskyttelse)**  **IF ONLY PIGS WERE HENS**  You have said no to battery hens – now the pig needs your help  BATTERY **NO**  PIGS **THANKS** |

**Q27c First, we would like to know if the add was displayed on your screen (a picture and a text)?**

1. Yes

2. No (jump to 33c)

**Q28c Have you seen this ad before, in newspapers, magazines, or on billboards on the street?**

1. Yes, definitely

2. Yes, I believe so

3. No, I don’t remember

4. No, definitely not

5. I’m not sure

**To which extend do you agree or disagree with the following statements about the campaign?**

**Q29c I have great sympathy for the campaign**

**Q30c The message in the campaign is misleading**

**Q31c The campaign is good because it says something central about the way pigs are treated in Danish farms**

1. Completely agree

2. Agree

3. Neither agree nor disagree

4. Disagree

5. Completely disagree

(Q32c is only presented if Q31c = ‘Completely agree’ or ‘Agree’)

**Q32c In your view, what does the campaign say about pig farming in Denmark?**

(Open-ended question)

Here is the next ad.

(In the table below, the original Danish poster is shown in the left-hand column, while an English translation is provided in the right-hand column.)

| **ORIGINAL POSTER (presented to respondents)** | **ENGLISH TRANSLATION** |
| --- | --- |
| 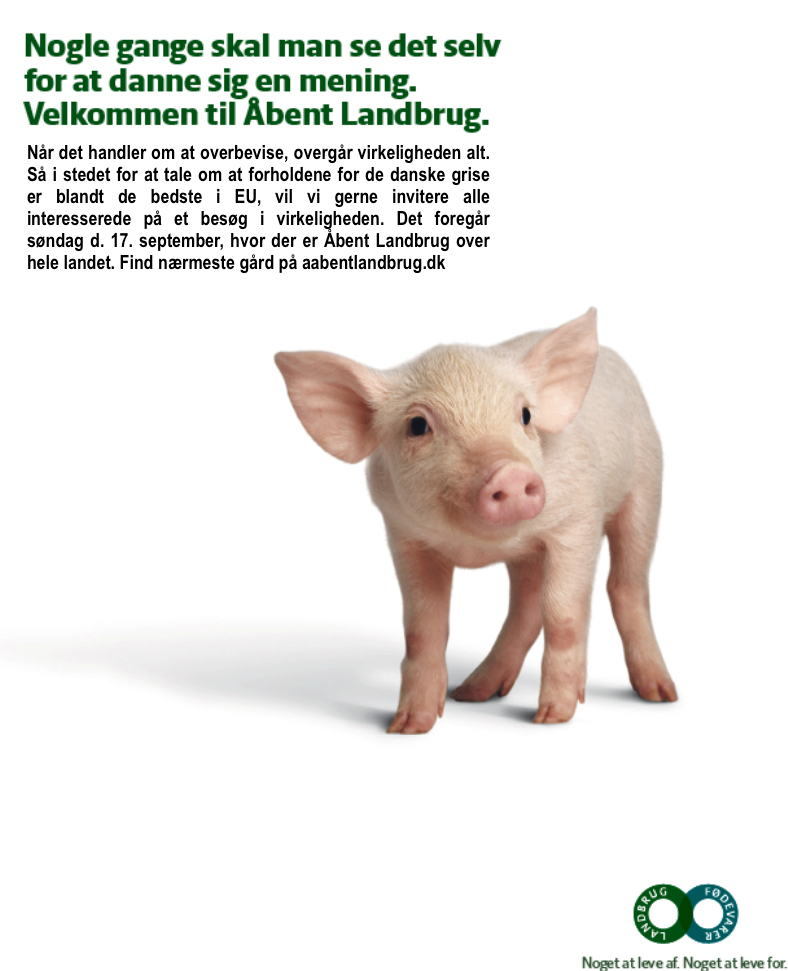 | **Sometimes you have to see it yourself to form an attitude. Welcome to Open Farm.**  When it comes to persuading, reality trumps everything else. So instead of just telling you that the conditions for pigs in Denmark are among the best in the EU, we would like to invite you to a visit into reality. Throughout the country, we have Open Farm on Sunday 17^th^ September. Find your nearest farm on aabentlandbrug.dk.  **Farm and Food** |

**Q33c First we would like to know if the add was displayed on your screen (a picture and a text)?**

1. Yes

2. No (jump to Q39)

**Q34c Have you seen this ad before, in newspapers, magazines, or on billboards on the street?**

1. Yes, definitely

2. Yes, I believe so

3. No, I don’t remember

4. No, definitely not

5. I’m not sure

**To which extend do you agree or disagree with the following statements about the campaign?**

**Q35c I have great sympathy for the campaign**

**Q36c The message in the campaign is misleading**

**Q37c The campaign is good because it says something central about the way pigs are treated in Danish farms**

1. Completely agree

2. Agree

3. Neither agree nor disagree

4. Disagree

5. Completely disagree

(Q38c is only presented if Q37c = ‘Completely agree’ or ‘Agree’)

**Q38c In your view, what does the campaign say about pig farming in Denmark?**

(Open-ended question)

**Block D questions**

Now, we would like to ask you about your perception of the messages in the following two ads that were used in recent campaign. You can see the first ad here.

(In the table below, the original Danish poster is shown in the left-hand column, while an English translation is provided in the right-hand column.)

| **ORIGINAL POSTER (presented to respondents)** | **ENGLISH TRANSLATION** |
| --- | --- |
| 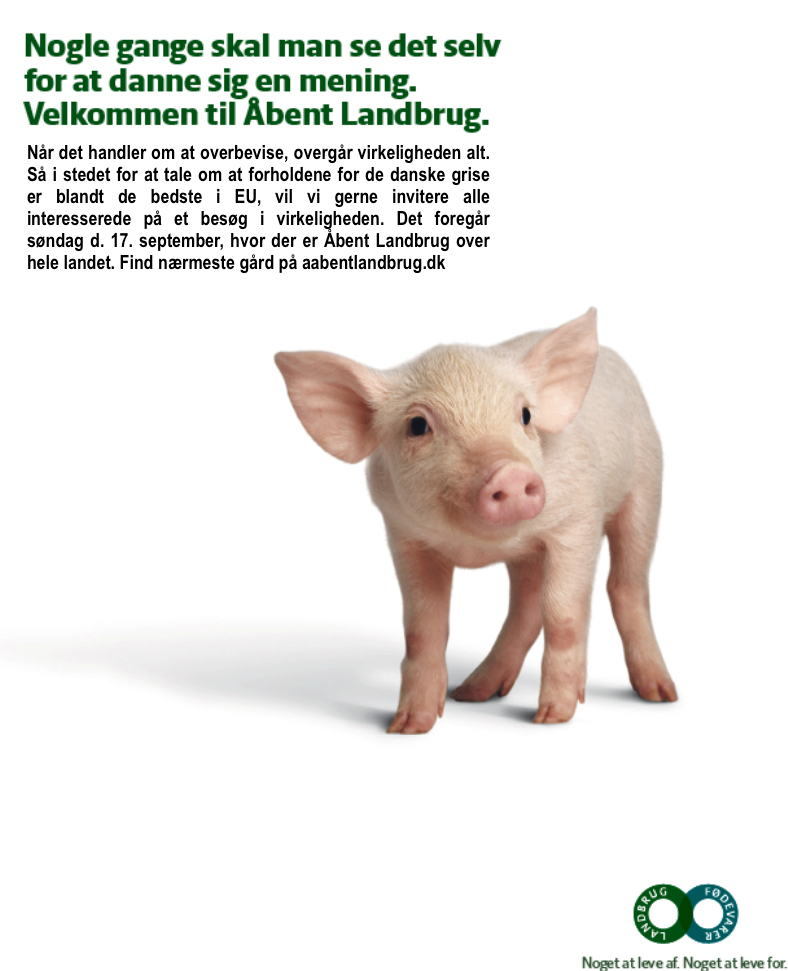 | **Sometimes you have to see it yourself to form an attitude. Welcome to Open Farm.**  When it comes to persuading, reality trumps everything else. So instead of just telling you that the conditions for pigs in Denmark are among the best in the EU, we would like to invite you to a visit into reality. Throughout the country, we have Open Farm on Sunday 17^th^ September. Find your nearest farm on aabentlandbrug.dk.  **Farm and Food** |

**Q27d First we would like to know if the add was displayed on your screen (a picture and a text)?**

1. Yes

2. No (jump to Q33d)

**Q28d Have you seen this ad before, in newspapers, magazines, or on billboards on the street?**

1. Yes, definitely

2. Yes, I believe so

3. No, I don’t remember

4. No, definitely not

5. I’m not sure

**To which extend do you agree or disagree with the following statements about the campaign?**

**Q29d I have great sympathy for the campaign**

**Q30d The message in the campaign is misleading**

**Q31d The campaign is good because it says something central about the way pigs are treated in Danish farms**

1. Completely agree

2. Agree

3. Neither agree nor disagree

4. Disagree

5. Completely disagree

(Q32d is only presented if Q31d = ‘Completely agree’ or ‘Agree’)

**Q32d In your view, what does the campaign say about pig farming in Denmark?**

(Open-ended question)

Here is the next ad.

(In the table below, the original Danish poster is shown in the left-hand column, while an English translation is provided in the right-hand column.)

| **ORIGINAL POSTER (presented to respondents)** | **ENGLISH TRANSLATION** |
| --- | --- |
|  | **Name of the NGO**  **(Dyrenes Beskyttelse)**  **IF ONLY PIGS WERE HENS**  You have said no to battery hens – now the pig needs your help  BATTERY **NO**  PIGS **THANKS** |

**Q33d First, we would like to know if the add was displayed on your screen (a picture and a text)?**

1. Yes

2. No (jump to Q39)

**Q34d Have you seen this ad before, in newspapers, magazines, or on billboards on the street?**

1. Yes, definitely

2. Yes, I believe so

3. No, I don’t remember

4. No, definitely not

5. I’m not sure

**To which extend do you agree or disagree with the following statements about the campaign?**

**Q35d I have great sympathy for the campaign**

**Q36d The message in the campaign is misleading**

**Q37d The campaign is good because it says something central about the way pigs are treated in Danish farms**

1. Completely agree

2. Agree

3. Neither agree nor disagree

4. Disagree

5. Completely disagree

(Q38d is only presented if Q37d = ‘Completely agree’ or ‘Agree’)

**Q38d In your view, what does the campaign say about pig farming in Denmark?**

(Open-ended question)

**Final questions**

**Q39 Do you or anyone else in the household have a companion animal?**

(All kinds of companion animals are included, e.g. rabbits, cats, and dogs)

1. Yes (jump to Q40)

2. No (jump to Q41)

**Q40 What kind of companion animal/animals is/are there in the household?**

(Multiple responses are allowed)

1. Hamster, guinea pig, rabbit, mouse or other rodents

2. Bird

3. Cat

4. Dog

5. Other animals

**Q41 Have you visited any of the following places in the past year?**

(Multiple responses are allowed)

1. Amusement and theme Parks (e.g. Tivoli, Legoland, BonBon-Land (Denmark))

2. ‘Summer amusement parks’ (e.g. Fårup Sommerland and Djurs Sommerland (Denmark) or other water parks)

3. Zoos or animal parks

4. Circuses

5. Aquariums (e.g. Danmarks Akvarium and Nordsøen Oceanarium (Denmark))

6. Science centres (e.g. Eksperimentarium and Randers Regnskov (Denmark))

7. No, none of these

**(Q42 & 43) How often do you eat the following bread toppings?**

Q42 Meat-topping

Q43 Fish-topping

1. More than twice a day

2. 1-2 times a day

3. 4-6 times a week

4. 1-3 times a week

5. Less often or never

**(Q44-47) How often do you eat hot meals with the following ingredients?**

Q44 Meat (beef, veal, pork or lamb)

Q45 Poultry (e.g. chicken, turkey or duck)

Q46 Fish

Q47 Vegetable or vegetarian dishes

1. More than once a day

2. 5-7 times a week

3. 3-4 times a week

4. 1-2 times a week

5. Less often or never

**Q48 Would you describe yourself as:**

1. Vegetarian: somebody who doesn’t eat meat, poultry, fish or seafood (jump to Q50)

2. Vegan: somebody who doesn’t eat meat, poultry, fish, seafood, dairy products or eggs (jump to Q50)

3. One who generally eats vegetarian food but occasionally, i.e. a maximum of 1-2 times a week or on special occasions, eat meat, poultry, fish or seafood (jump to Q49)

4. None of these (jump to Q49)

**Q49 To which extend do you agree with the following statement: I attempt to only eat meat from livestock productions where certain animal welfare standards have been met.**

1. Completely agree

2. Agree

3. Neither agree nor disagree

4. Disagree

5. Completely disagree

**Q50 Do you work within one or more of these job categories?**

(Multiple responses are allowed)

1. Farmer or farm assistant

2. Consultant/advisor/vet in livestock productions, slaughterhouses or meat packing companies

3. Strategic, economic or political work concerning livestock productions

4. Butcher, slaughterhouse worker or livestock haulier

5. Work in a meat packing company, meat retail store/butcher's shop

6. No, none of these

**Q51 if elections were held tomorrow, which political party would you vote for?**

(Randomised, the following list displays the names of the most significant parties in Denmark)

1. Socialdemokratiet

2. Radikale Venstre

3. Det Konservative Folkeparti

4. Socialistisk Folkeparti

5. Dansk Folkeparti

6. Alternativet

7. Nye borgerlige

8. Kristendemokraterne

9. Venstre

10. Liberal Alliance

11. Enhedslisten

12. Other

13. I wouldn’t vote

14. I don’t know/prefer not to answer
